# Supplementary material for: Cyclooxygenase-Dependent Tumor Growth through Evasion of Immunity
Source: Cell. 2015 Sep 10;162(6):1257–70. doi: 10.1016/j.cell.2015.08.015 (PMC4597191; doi:10.1016/j.cell.2015.08.015)
Supplement: Document S1. Supplemental Experimental Procedures [file mmc1.pdf]

Cell

Supplemental Information

Cyclooxygenase-Dependent Tumor Growth through Evasion of Immunity

Santiago Zelenay, Annemarthe G. van der Veen, Jan P. Böttcher, Kathryn J. Snelgrove, Neil Rogers, Sophie E. Acton, Probir Chakravarty, Maria Romina Girotti, Richard Marais, Sergio A. Quezada, Erik Sahai, and Caetano Reis e Sousa

Supplemental Experimental Procedures

Mice. C57BL/6, *Rag1*$^{-/-}$, *Tap1*$^{-/-}$, *H2-Aa*$^{-/-}$ (*I-A*$\alpha^{-/-}$), *Batf3*$^{-/-}$ (kind gift from Kenneth M. Murphy, Washington University St. Louis), *Ifnar1*$^{-/-}$, Balb/c and nude mice were bred at Cancer Research UK in specific pathogen-free conditions.

Cancer cell lines. The MCA fibrosarcoma cell line was a kind gift from Robert D. Schreiber (Washington University St. Louis). COX-2 knockdown was performed using five non-overlapping shRNA hairpins targeting the coding sequence of murine *Ptgs2* (Ptgs2, NM_011198.2) selected via the RNAi Consortium of the Broad Institute (http://www.broadinstitute.org/rnai/public/). Three hairpins targeting eGFP were chosen as controls. Appropriate DNA oligonucleotides encoding these hairpins were cloned into the AgeI/EcoRI site of the lentiviral plasmid pLKO.1neo (obtained from Dr. S. Stewart via Addgene, plasmid 13425). Lentivirus was produced according to instructions provided by the Broad Institute (http://www.broadinstitute.org/rnai/public/resources/protocols). For generation of *Ptgs2*$^{-/-}$ Braf^{V600E} cells the *Ptgs2* sequence AGATGACTGCCCAACTCCCA**TGG** was targeted using the oligos Forward (Fw) CACCGGATGACTGCCCAACTCCCA and Reverse (Rv) AAACTGGGAGTTGGGCAGTCATCC. For sgRNAs 1 *Ptgs1/Ptgs2*$^{-/-}$ Braf^{V600E} cells the *Ptgs1* sequence TTACTATCCGTGCCAGAACC**AGG** was targeted using the oligos Fw CACCGTACTATCCGTGCCAGAACC and Rv AAACGGTTCTGGCACGGATAGTAC and the *Ptgs2* sequence TCCAATCCATGTCAAAACCG**TGG** using oligos Fw CACCGCCAATCCATGTCAAAACCG and Rv AAACCGGTTTTGACATGGATTGGC. For sgRNAs 2 *Ptgs1/Ptgs2*$^{-/-}$ cells the *Ptgs1* sequence CCGAGAAGTACTCATGCGCC**TGG** was

targeted using the oligos Fw CACCGCGAGAAGTACTCATGCGCC and Rv AAACGGCGCATGAGTACTTCTCGC and the *Ptgs2* sequence TCCAATCCATGTCAAAACCG**TGG** using oligos Fw CACCGCCAATCCATGTCAAAACCG and Rv AAACCGGTTTTGACATGGATTGGC. For sgRNAs 3 *Ptgs1/Ptgs2*$^{-/-}$ cells the *Ptgs1* sequence TTACTATCCGTGCCAGAACC**AGG** was targeted using the oligos Fw CACCGTACTATCCGTGCCAGAACC and Rv AAACGGTTCTGGCACGGATAGTAC and the *Ptgs2* sequence AGATGACTGCCCAACTCCCA**TGG** using oligos Fw CACCGGATGACTGCCCAACTCCCA and Rv AAACTGGGAGTTGGGCAGTCATCC. For *Ptgs2*$^{-/-}$ Nras^{G12D} melanoma cells and *Ptgs2*$^{-/-}$ CT26 cells the *Ptgs2* sequence TCCAATCCATGTCAAAACCG**TGG** was targeted using the oligos Fw CACCTCCAATCCATGTCAAAACCG and Rv AAACCGGTTTTGACATGGATTGGA. For *Ptgs1/Ptgs2*$^{-/-}$ 4T1 cells the *Ptgs1* sequence TTACTATCCGTGCCAGAACC**AGG** was targeted using the oligos Fw CACCGTACTATCCGTGCCAGAACC and Rv TCCAATCCATGTCAAAACCG**TGG** and the *Ptgs2* sequence using the oligos Fw CACCTCCAATCCATGTCAAAACCG and Rv AAACCGGTTTTGACATGGATTGGA.

For *Pges*$^{-/-}$ (*mPGES-1/mPGES-2*$^{-/-}$) Braf^{V600E} cells the *mPGES-1* sequence GGTCATCAAGATGTACGCGG**TGG** was targeted using the oligos Fw CACCGGTCATCAAGATGTACGCGG and Rv AAACCCGCGTACATCTTGATGACC and the *mPGES-2* sequence CCAGGCGGCGCGCCTTTCGT**GGG** using the oligos Fw CACCGCAGGCGGCGCGCCTTTCGT and Rv AAACCTTGCGGACCGCGGCGCCCC. Oligonucleotides corresponding to these guide sequences (PAM motif in bold) were cloned into the BbsI site of pX330, a bicistronic expression vector encoding both Cas9 and the sgRNA.

Plasmid pX330 was obtained from the Zhang lab via Addgene (plasmid 42230). $Braf^{V600E}$ cells were co-transfected in a 6-well plate using 1 μg pX330 and 1 μg pEGFP-C1 (Clontech) per well using Lipofectamine 2000 (Life technologies) according to the manufacturer's instructions. Twenty-four to 48 h post-transfection, GFP-positive cells were FACS sorted and replated at limiting dilution in order to pick individual colonies. Knockdown of COX-2 and ablation of COX-1 and/or COX-2 were also verified by immunoblotting using COX-1 and COX-2 specific antibodies (Cell Signaling). For determination of PGE_2 levels in vivo a fixed number of total tumor cell suspension was freeze-thawed and re-suspended in 100 μl. The concentration of PGE_2 in CM or in vivo was determined by ELISA (Cayman chemical). The proliferative capacity of cells was assessed using a non-radioactive cell proliferation assay (Promega) following manufacturer's instructions. The Braf inhibitor, PLX4720, was from Stratech and the MEK inhibitor, PD184352 from LC Labs. Anti-ERK and anti-phospho-ERK antibodies were from Cell Signaling. Anti-p97 antibody was from Fitzgerald Industries.

FACS analysis. For analysis of tumor infiltrating DCs, tumors were collected, cut into small pieces and digested with Collagenase IV (200 U/ml) and DNase I (0.2 mg/ml) for one hour at 37°C, washed with FACS buffer (PBS containing 2% FCS, 2 mM EDTA and 0.02% sodium azide) and pelleted. The following antibodies were purchased from BD Biosciences: anti-CD86-Fluorescein isothiocyanate (FITC) (clone GL1), anti-CD40-Phycoerythrin (PE) (clone 3/23), anti-CD16/32 (clone 2.4G2; Fc block) and anti-CD19-V450 (clone 1D3). The following antibodies were purchased from eBioscience: anti-MHCII I-A/I-E-APC-eFluor780 (clone M5/114.15.2), anti-CD11b-Alexa Fluor 700 or eFluor 605Nanocrystal (clone M1/70), anti-Ly6G-Alexa Fluor 700 (clone RB6-8C5) and anti-CD103 APC (clone 2E7). Anti-CD45.2-

PECy7 (clone 104) and anti-CD11c-PerCP/Cy5.5 (clone N418),were from Biolegend. Propidium iodide or Dapi were added to the final suspension to exclude dead cells before acquisition on a FACS-Calibur or LSR Fortessa (BD Biosciences) and analysis performed with Flowjo (Tree Star, Ashland, OR) software. Live cell counts were calculated from the acquisition of a fixed number of 10 μm latex beads (Coulter) mixed with a known volume of unstained cell suspension. Ex vivo intracellular IL-12 p40 staining was performed on isolated cells 6 hours following an intravenous injection of Brefeldin A (10 mg/g of body weight, Sigma-Aldrich). Tumor digestion was performed in presence of Brefeldin A (5 μg/ml). Cells were then stained for surface markers, fixed, permeabilized and then stained with anti-mouse PE-coupled anti-IL-12/23 p40 Ab (ebioscience, clone C17.8).

In vitro culture. Heat inactivation was performed by incubating CM before addition to DC cultures at 95°C for 20'. Nuclease treatment consisted in incubation of CM in presence of 2.5 Units/μl benzonase (Sigma) at 37°C for 30'. Synthetic PGE_2 was from Sigma. Capture anti-IL-12/23 p40 (BD Biosciences) and detection biotinylated anti-IL-12/23 p40 (BD Biosciences) antibodies were used for ELISA. Mouse TNF, MIP1α, IL-6, CXCL1, G-CSF, IL-1β, IL-10 and RANTES cytometric bead array flex sets were from BD Biosciences.

Quantitative PCR. Expression of *Ptgs2, Cd86, Tbx21, Ifng, Cxcl10, Cxcl11, Inos, Arg1, E-cadherin* and *Gas3* was determined by quantitative PCR using Invitrogen SYBR Green Fast master mix according to the manufacturer's instructions. Primers used: *Ptgs2*: Fw 5'-GAAGTGCCAATCCCCTAGCAA and Rv 5'- ATACCTGGAAGAACTTGATGCCC; *Cd86*: Fw 5'-ACAAAAAAAGCCACCCACAG and Rv 5'-ACGTGCAGGTCAAATTTATGC;

Tbx21: 5'-CAACAACCCCTTTGCCAAAG and Rv 5'- TCCCCCAAGCAGTTGACAGT; *Ifng*: Fw 5'-GGATGCATTCATGAGTATTGC and Rv 5'-CCTTTTCCGCTTCCTGAGG; *Cxcl10*: Fw 5'- TCAAGCCATGGTCCTGAGACAA and Rv 5'-CGCACCTCCACATAGCTTACAG; *Cxcl11*: Fw 5'-CCTGGGAACGTCTGACTGTG and Rv 5'- TCTGCAGCCTGGTAATACGTG; *Inos*: Fw 5'-CAGCTGGGCTGTACAAACCTT and Rv 5'-CATTGGAAGTGAAGCGTTTCG; Arg1: Fw 5'-GAACACGGCAGTGGCTTTAAC and Rv 5'-TGCTTAGCTCTGTCTGCTTTGC; E-cadherin: Fw 5'-ACTTGGGGACAGCAACATCA and Rv 5'-GGGTTTAAATCGGCCAGCAT; Gas3: Fw 5'-GTAATGGACACACGACTGATC and Rv 5'-GGAGTAGTCAGTGTTGACATG. HPRT: Fw 5'-TCAGTCAACGGGGGACATAAA and Rv 5'-GGGGCTGTACTGCTTAACCAG as normalization control was used to assess relative gene expression. Expression of *Il6* (Mm00446190_m1), *Cxcl1* (Mm04207460_m1), *Il12b* (Mm99999067_m1), *Tnf* (Mm00443258_m1), *Ifit1* (Mm00515153_m1) and *Ifit2* (Mm00492606_m1) was determined by relative standard curve method using Taqman reagents according to manufacturer's instructions and normalized to GapDH (Mm99999915_g1) expression (Applied Biosystems).

Human microarray dataset analysis. Data were analyzed using Bioconductor 2.13 running on R 3.0.2 (available from www.R-project.org). Probeset expression measures were calculated using the Affymetrix package's Robust Multichip Average (RMA) default method. The GSE3189 dataset comprised of 45 melanoma samples and were ranked from lowest to highest using COX-2 (PTGS2) expression. The top and bottom 10 samples (~20%) were extracted from the dataset and used to look for enrichment of gene sets using Gene set enrichment analysis (GSEA)(Subramanian et al., 2005).
