# Supplementary material for: Cyclooxygenase-Dependent Tumor Growth through Evasion of Immunity
Source: Cell. 2015 Sep 10;162(6):1257–70. doi: 10.1016/j.cell.2015.08.015 (PMC4597191; doi:10.1016/j.cell.2015.08.015)
Supplement: Document S2. Article plus Supplemental Information [file mmc2.pdf]

Cell

Article

Cyclooxygenase-Dependent Tumor Growth through Evasion of Immunity

Graphical Abstract

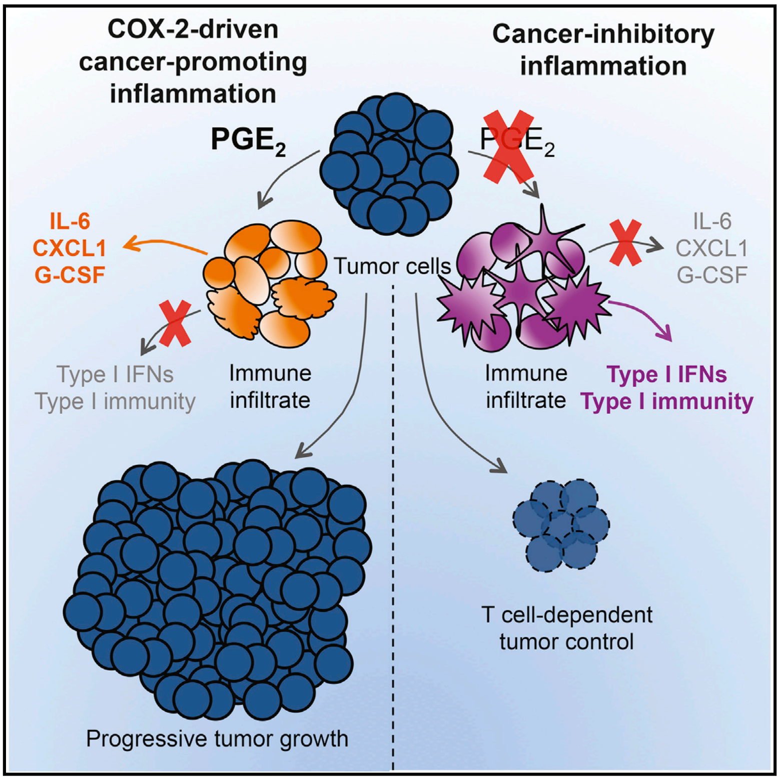

Highlights

- Cyclooxygenase in tumors induces PGE_2 that subverts myeloid cell function
- COX ablation in tumors enables immune control
- COX inhibition synergizes with checkpoint blockade therapy
- A COX inflammatory signature is conserved across mouse and human cancer biopsies

Authors

Santiago Zelenay, Annemarthe G. van der Veen, Jan P. Böttcher, ..., Sergio A. Quezada, Erik Sahai, Caetano Reis e Sousa

Correspondence

santiago.zelenay@cruk.manchester.ac.uk (S.Z.),
caetano@crick.ac.uk (C.R.S.)

In Brief

Cyclooxygenase-driven prostaglandin E2, produced by a variety of tumors, drives malignant growth through successful evasion of type I interferon and/or T-cell-dependent tumor elimination. A remarkable synergy between cyclooxygenase inhibitors and checkpoint blockade immunotherapy results in tumor eradication.

Zelenay et al., 2015, Cell *162*, 1257–1270
September 10, 2015 ©2015 The Authors
http://dx.doi.org/10.1016/j.cell.2015.08.015

Cyclooxygenase-Dependent Tumor Growth through Evasion of Immunity

Santiago Zelenay,[1,6,*] Annemarthe G. van der Veen,[1] Jan P. Böttcher,[1] Kathryn J. Snelgrove,[1] Neil Rogers,[1] Sophie E. Acton,[1] Probir Chakravarty,[2] Maria Romina Girotti,[3] Richard Marais,[3] Sergio A. Quezada,[4] Erik Sahai,[5] and Caetano Reis e Sousa[1,*]
[1]Immunobiology Laboratory, The Francis Crick Institute, Lincoln's Inn Fields Laboratory, 44 Lincoln's Inn Fields, London WC2A 3LY, UK
[2]Bioinformatics, The Francis Crick Institute, Lincoln's Inn Fields Laboratory, 44 Lincoln's Inn Fields, London WC2A 3LY, UK
[3]Molecular Oncology Group, Cancer Research UK Manchester Institute, The University of Manchester, Manchester M20 4BX, UK
[4]Cancer Immunology Unit, Research Department of Haematology, University College London Cancer Institute, London WC1E 6DD, UK
[5]Tumor Cell Biology Laboratory, The Francis Crick Institute, Lincoln's Inn Fields Laboratory, 44 Lincoln's Inn Fields, London WC2A 3LY, UK
[6]Present address: Cancer Inflammation and Immunity Group, Cancer Research UK Manchester Institute, The University of Manchester, Manchester M20 4BX, UK
*Correspondence: santiago.zelenay@cruk.manchester.ac.uk (S.Z.), caetano@crick.ac.uk (C.R.S.)
http://dx.doi.org/10.1016/j.cell.2015.08.015
This is an open access article under the CC BY license (http://creativecommons.org/licenses/by/4.0/).

SUMMARY

The mechanisms by which melanoma and other cancer cells evade anti-tumor immunity remain incompletely understood. Here, we show that the growth of tumors formed by mutant $Braf^{V600E}$ mouse melanoma cells in an immunocompetent host requires their production of prostaglandin E2, which suppresses immunity and fuels tumor-promoting inflammation. Genetic ablation of cyclooxygenases (COX) or prostaglandin E synthases in $Braf^{V600E}$ mouse melanoma cells, as well as in $Nras^{G12D}$ melanoma or in breast or colorectal cancer cells, renders them susceptible to immune control and provokes a shift in the tumor inflammatory profile toward classic anti-cancer immune pathways. This mouse COX-dependent inflammatory signature is remarkably conserved in human cutaneous melanoma biopsies, arguing for COX activity as a driver of immune suppression across species. Pre-clinical data demonstrate that inhibition of COX synergizes with anti-PD-1 blockade in inducing eradication of tumors, implying that COX inhibitors could be useful adjuvants for immune-based therapies in cancer patients.

INTRODUCTION

Inflammation has emerged as a major factor promoting cancer development (Coussens et al., 2013; Grivennikov et al., 2010; Mantovani et al., 2008; Rakoff-Nahoum and Medzhitov, 2009). Tumor-promoting inflammation is characterized by the presence of sub-types of neutrophils, macrophages, dendritic cells (DCs), and T lymphocytes that support cancer progression (Balkwill et al., 2005; Coussens et al., 2013; Mantovani et al., 2008). Mediators secreted by these cells that directly or indirectly promote cancer cell growth include cytokines, chemokines, and growth factors, such as VEGF-A, CSFs, IL-1, IL-6, IL-8, or CXCL1 (Balkwill et al., 2005; Coussens et al., 2013). Yet inflammation can also have cancer-inhibitory effects (Coussens et al., 2013; Mantovani et al., 2008), in part by favoring immune attack (Vesely et al., 2011). Indeed, in most mouse and human cancers, the presence of immune cells, such as cytotoxic T cells and DCs (in particular, the Batf3-dependent $CD103^{+}$ sub-type), or of inflammatory mediators, such as type I interferons (IFNs), IFN-γ, and IL-12, is associated with good prognosis (Fridman et al., 2012; Gajewski et al., 2013; Vesely et al., 2011). Notably, several "immune checkpoint blockade" therapies aimed at unleashing the anti-cancer potential of tumor-specific T cells have recently shown great promise (Page et al., 2014; Sharma and Allison, 2015). These observations suggest that cancer cells do not pass unnoticed by the immune system but actively evade anti-tumor immunity.

In line with the above, tumors arising in immunosufficient hosts are commonly poorly immunogenic as a consequence of immunoediting (Schreiber et al., 2011). Reduced tumor immunogenicity can be a "recessive" consequence of downregulation of antigen-presenting MHC molecules or loss of antigens that serve as targets for T cell-mediated control (DuPage et al., 2012; Matsushita et al., 2012). Loss of immunogenicity can also be due to blockade of T cell access to tumor cell targets, recruitment of suppressive cells, and/or production of immunosuppressive factors (Joyce and Fearon, 2015). The latter can act in part by dampening production of type I interferons, IL-12, and other factors that are required for priming or restimulating anti-tumor T cells and for sustaining T cell-independent anti-tumor immunity (Dunn et al., 2005; Vesely et al., 2011). Unlike recessive mechanisms of immunoediting, immunosuppressive factors act in a dominant fashion and therefore offer a unique opportunity for immune therapy intervention so long as the antigenic determinants for tumor rejection have not been lost.

Inflammatory mediators can be produced by the stroma, by tumor-infiltrating leukocytes, or directly by the cancer cells themselves. Prominent among tumor-sustaining mediators is prostaglandin E2 (PGE_2), a prostanoid lipid associated with enhancement of cancer cell survival, growth, migration, invasion, angiogenesis, and immunosuppression (Wang and Dubois, 2010). Cyclooxygenase (COX)-1 and 2, enzymes critical for the production of PGE_2, are often overexpressed in colorectal,

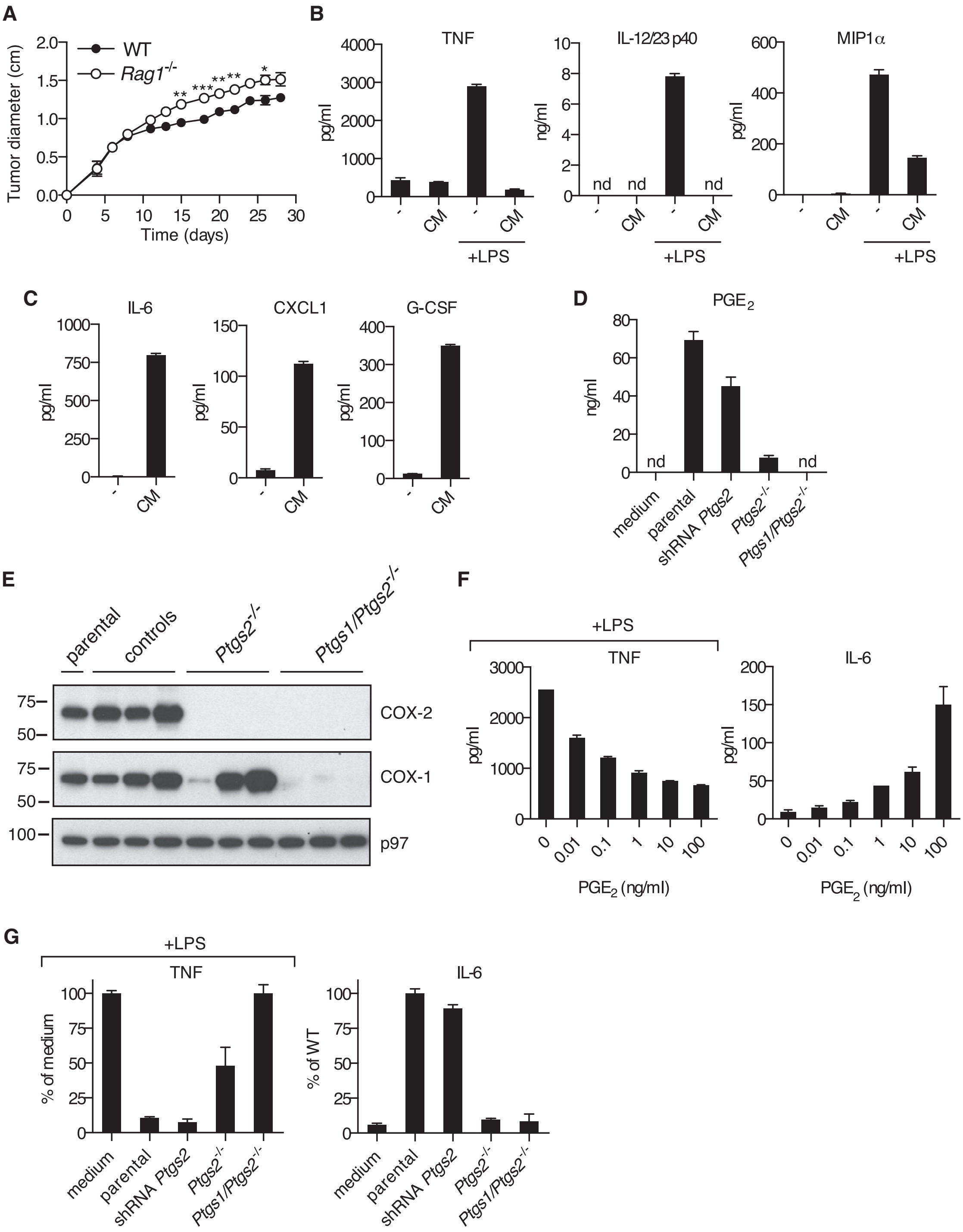

Figure 1. COX-1- and COX-2-Dependent Tumor-Derived Prostanoids Modulate Myeloid Cells

(A) Growth of $Braf^{V600E}$ cells following implantation into WT and *Rag1*$^{-/-}$ mice. Data are presented as average tumor diameters ± SEM and are representative of three independent experiments with three to five mice per group. Tumor growth profiles were compared using two-way ANOVA. $^{*}p < 0.05$, $^{**}p < 0.01$, $^{***}p < 0.001$.

(B and C) BMMCs were cultured in the presence or absence of CM from $Braf^{V600E}$ cells with or without LPS (100 ng/ml). The concentration of TNF, IL-12/23 p40, and MIP1α (B) or IL-6, CXCL1, and G-CSF (C) in supernatants was determined after overnight culture.

(D) $Braf^{V600E}$ cells unmodified (parental), control, stably expressing a *Ptgs2*-specific targeting shRNA construct, *Ptgs2*$^{-/-}$, or *Ptgs1/Ptgs2*$^{-/-}$ were cultured to confluency, and the concentration of PGE_2 in the supernatant was determined by ELISA.

(E) Immunoblot of COX-2 and COX-1 in parental $Braf^{V600E}$ cells and three independent control, *Ptgs2*$^{-/-}$, or *Ptgs1/Ptgs2*$^{-/-}$ clones generated by CRISPR/Cas9-mediated genome engineering using distinct sets of sgRNAs targeting different regions of the *Ptgs1* and *Ptgs2* loci. p97 served as a loading control.

(legend continued on next page)

breast, stomach, lung, and pancreatic cancers (Dannenberg and Subbaramaiah, 2003; Wang and Dubois, 2010). Here, we identify tumor-derived COX activity in a mouse melanoma driven, as in human, by an oncogenic mutation in Braf, as the key suppressor of type I IFN- and T cell-mediated tumor elimination and the inducer of an inflammatory signature typically associated with cancer progression. COX-dependent immune evasion was also critical for tumor growth in other melanoma, colorectal, and breast cancer models. Notably, tumor immune escape could be reversed by a combination of immune checkpoint blockade and administration of COX inhibitors, suggesting that the latter may constitute useful additions to the arsenal of anti-cancer immunotherapies.

RESULTS

$Braf^{V600E}$ Melanoma Cell Supernatants Have Immunomodulatory Effects on Myeloid Cells

In order to identify immune evasion mechanisms operative in melanoma, we used a transplantable tumor cell line established from a $Braf^{+/LSL\text{-}V600E}$;Tyr::CreERT2$^{+/o}$;p16$^{INK4a-/-}$ mouse (Dhomen et al., 2009) (henceforth, $Braf^{V600E}$ cells). We reasoned that such cells, isolated from a genetically engineered cancer-prone mouse bearing an intact immune system, are likely to possess key attributes that allow them to escape immune control in the original host. Indeed, underscoring their poor immunogenicity, $Braf^{V600E}$ melanoma cells formed progressively growing tumors upon implantation into wild-type (WT) mice, and this was only marginally enhanced in T- and B-cell-deficient *Rag1*$^{-/-}$ mice (Figure 1A). We tested whether the poor immunogenicity of $Braf^{V600E}$ cells could result from compromised or subverted activation of antigen-presenting cells, including dendritic cells (DCs) and monocyte-derived cells. We cultured mouse bone marrow-derived mononuclear cells (BMMCs), a mixed population of DCs and monocyte-derived macrophages (Helft et al., 2015), with conditioned medium (CM) from $Braf^{V600E}$ cells in the presence or absence of a strong innate immune stimulus, lipopolysaccharide (LPS). Remarkably, LPS-induced production of TNF, IL-12/23p40, and MIP1α by BMMCs was strongly inhibited by CM from $Braf^{V600E}$ cells (Figure 1B). Moreover, addition of CM alone induced a distinct set of proinflammatory mediators, including IL-6, CXCL1, and G-CSF (Figure 1C). The latter, as well as IL-1β, IL-10, and RANTES, were also induced by LPS, but CM, if anything, enhanced their accumulation (Figure S1A). Thus, tumor-derived secreted factors subvert the normal pattern of myeloid cell-driven inflammation.

Cyclooxygenase-Dependent Prostanoids Account for the Immunomodulatory Effects of $Braf^{V600E}$ Tumors on Myeloid Cells

Neither heat inactivation (to denature proteins) nor benzonase treatment (to degrade nucleic acids) impacted the ability of tumor CM to promote IL-6 production or inhibit LPS-dependent induction of TNF by BMMCs (Figure S1B). We therefore investigated whether the immunomodulatory factor might be a lipid. The ability to inhibit IL-12 p40 production was reminiscent of the effects of the prostanoid PGE_2 (Kalinski, 2012), which we found in high amounts in CM from $Braf^{V600E}$ melanoma cells (Figure 1D). These cells also expressed cyclooxygenase (COX)-1 and -2, two enzymes critical for prostanoid synthesis (Figure 1E). Treatment with a Braf or a MEK inhibitor led to reduced COX-2 protein and PGE_2 secretion, indicating that COX-2 expression in $Braf^{V600E}$ cells was dependent on active RAF/MEK signaling (Figure S2).

Addition of synthetic PGE_2 to BMMCs mimicked the effect of CM (Figure 1F). To assess the importance of COX-2-derived prostanoids, we targeted the *Ptgs2* gene (encoding COX-2) in $Braf^{V600E}$ melanoma cells with several small hairpin RNAs (shRNAs). Although COX-2 expression was clearly diminished (Figure S3), the concentration of PGE_2 in CM from these cells was only modestly reduced and the modulatory effect of CM on BMMCs was unchanged (Figures 1D and 1G). As residual COX-2 expression could account for these observations, we resorted to CRISPR/Cas9 technology to generate *Ptgs2*$^{-/-}$ (COX-2-deficient) $Braf^{V600E}$ melanoma cells (Figure 1E). Production of PGE_2 by *Ptgs2*$^{-/-}$ CRISPR-targeted clones was greatly decreased (Figure 1D), and their CM was no longer able to inhibit LPS-mediated TNF production or to induce IL-6 secretion by BMMCs (Figure 1G). These data demonstrate that COX-2 expression largely accounts for the myeloid cell modulatory properties of CM from $Braf^{V600E}$ melanoma cells. Of note, in agreement with previous reports (Dannenberg and Subbaramaiah, 2003), we found PGE_2 in CM from many, but not all, mouse cancer cell lines, including 4T1 breast cells, CT26 colorectal cells, a line derived from a $Nras^{G12D}$-driven melanoma-bearing mouse (Pedersen et al., 2013), and a methylcholanthrene-induced fibrosarcoma (Matsushita et al., 2012) (Figure S4).

COX Activity in $Braf^{V600E}$ Cells Shifts the Inflammatory Profile at the Tumor Site

To test the effect of tumor-derived prostanoids in vivo, we inoculated WT mice with parental or *Ptgs2*$^{-/-}$ $Braf^{V600E}$ cells and assessed the expression of an array of inflammatory and immune mediators in whole-tumor biopsies of comparable size (Figure 2A), containing immune-infiltrating cells, at 4 days post-implantation. In agreement with the effects seen in vitro, loss of COX-2 expression by tumor cells led to a significant decrease in expression of IL-6 or CXCL1 in vivo (Figures 2B and 2C). In contrast, several mRNAs encoding known anti-tumor immune mediators or reflective of anti-tumor type I immunity, including IFN-γ, T-bet, CXCL10, and IL-12 (Vesely et al., 2011), were markedly increased in *Ptgs2*$^{-/-}$ melanomas (Figures 2B and 2D).

(F) BMMCs were cultured in the presence of increasing amounts of synthetic PGE_2 plus or minus LPS (100 ng/ml). The concentration of TNF (+LPS) and IL-6 (no LPS) in the supernatant was determined after overnight culture.

(G) BMMCs were cultured as in (B) or (C) in presence of CM from the indicated $Braf^{V600E}$ melanoma cell lines. The concentration of TNF after overnight culture is expressed relative to the concentration of TNF in the supernatant of BMMCs cultured in presence of LPS without any CM (% of medium). The concentration of IL-6 is expressed relative to the concentration of IL-6 in the supernatant of BMMCs cultured with CM from parental $Braf^{V600E}$ cells (% of parental). nd, not detected.

See also Figures S1, S2, S3, and S4.

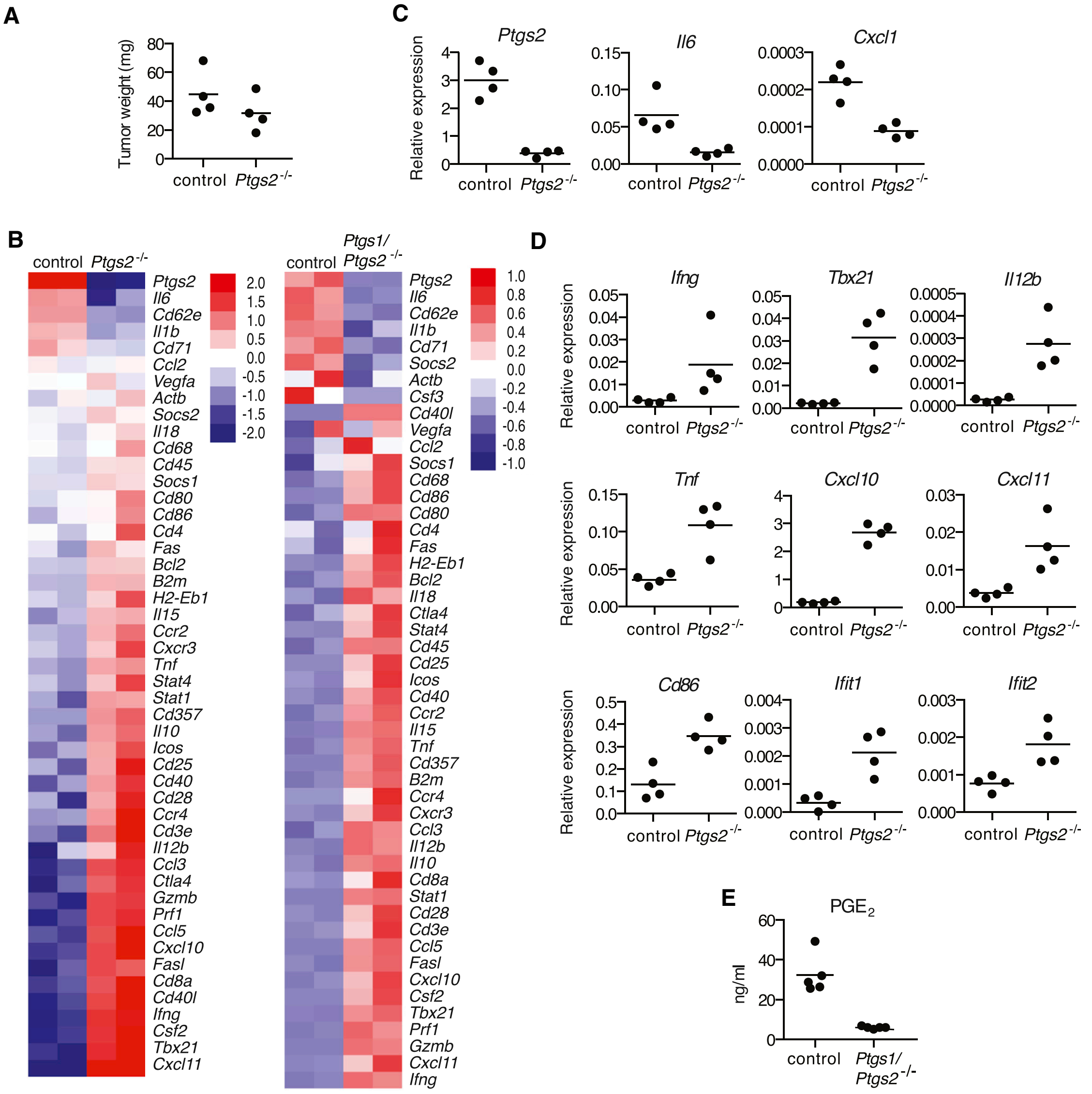

Figure 2. Genetic Ablation of COX in Braf^{V600E} Cells Shifts the Tumor Inflammatory Profile

(A–E) WT mice were inoculated with 10^6 control, *Ptgs2*$^{-/-}$, or *Ptgs1/Ptgs2*$^{-/-}$ Braf^{V600E} cells. 4 days later, the expression of an array of immune-associated genes was determined by qPCR in whole-tumor homogenates.

(A) Tumor weight at the time of harvest is shown.

(B) Heatmaps for a selected list of genes show log2 ΔCT values normalized to *hprt* of two biological replicates for each value. The genes are ordered from highest to lowest by fold change in control relative to *Ptgs2*$^{-/-}$ or *Ptgs1/Ptgs2*$^{-/-}$ samples.

(C and D) Relative expression of each gene normalized to *hprt*.

(E) Concentration of PGE_2 in lysates from 10^5 total tumor cells. Each dot represents one independent tumor.

See also Figure S5.

Likewise, mRNA levels of *Ifit1* and *Ifit2*, two type I IFN-stimulated genes (ISGs), were also elevated in *Ptgs2*$^{-/-}$ tumors (Figure 2D), indicative of enhanced type I interferon (IFN-α/β) signaling, which is central to immune-mediated tumor control (Gajewski et al., 2013; Vesely et al., 2011). We failed to detect a reduction in the expression of type 2 cytokines, such as IL-4, IL-5, or IL-13, or markers associated with M2 macrophage polarization, such as iNOS, arginase I, Gas-3, or E-cadherin (Figure S5; data not shown), despite the fact that they have been reported to be induced by prostanoids within tumors (Wang and Dubois, 2010). Also, we did not detect decreased expression of IL-10 (Figure 2B), an anti-inflammatory cytokine that has been suggested to mediate many of the immunosuppressive effects of COX-2 (Kalinski, 2012).

Even though COX-2 activity accounted for more than 90% of PGE_2 production by Braf^{V600E} cells, we still detected low levels of PGE_2 and some degree of BMMC modulatory activity in supernatants from *Ptgs2*$^{-/-}$ cells, particularly from clones that

stochastically displayed higher COX-1 expression (Figures 1D and 1G; data not shown). To fully eliminate COX activity and avoid potential in vivo selection for cells that compensate for COX-2 deficiency by upregulating COX-1, we generated COX-1 and COX-2 doubly deficient Braf^{V600E} cells (*Ptgs1/Ptgs2*$^{-/-}$ cells; Figure 1E). These cells fully lacked the ability to produce PGE_2 and did not modulate the activity of BMMCs in vitro (Figures 1D and 1G). Tumors formed by *Ptgs1/Ptgs2*$^{-/-}$ cells displayed markedly reduced global PGE_2 levels, indicating a dominant role for tumor-derived over stroma-derived PGE_2 in vivo (Figure 2E). Importantly, as for *Ptgs2*$^{-/-}$ singly deficient cells, a clear shift in the inflammatory profile toward increased expression of anti-tumor immune mediators was seen in tumors formed by *Ptgs1/Ptgs2*$^{-/-}$ doubly deficient cells (Figure 2B). We conclude that Braf^{V600E} melanoma-derived prostanoids drive the expression of multiple tumor-promoting cytokines and growth factors in the local tumor microenvironment, while preventing type I immunity and other anti-tumor immune effector pathways, including those controlled by type I IFNs.

COX Expression in Braf^{V600E} Cells Prevents CD103$^+$ DC Accumulation and Activation in Tumors

DCs, especially the Batf3-dependent sub-family characterized by CD8α and/or CD103 expression, are essential for anti-cancer immune responses (Diamond et al., 2011; Fuertes et al., 2011; Hildner et al., 2008). We therefore assessed the impact of tumor-specific COX ablation on the prevalence and activation status of DCs at the tumor site, focusing on the CD103$^+$ subset. Despite being only a minority of DCs, CD103$^+$ DCs were selectively absent from COX-competent tumors (Figure 3A). Moreover, the fraction of intratumoral CD103$^+$ DCs producing IL-12 p40 was higher in COX-deficient tumors (Figure 3B). Finally, CD103$^+$ DCs, as well as CD103$^-$ CD11b$^+$ DCs, displayed higher levels of costimulatory molecules in tumors formed by *Ptgs1/Ptgs2*$^{-/-}$ cells (Figure 3B). Thus, tumor-derived prostanoids impair accumulation of Batf3-dependent CD103$^+$ DCs within tumors and suppress their activation, including IL-12-producing activity.

Genetic Ablation of COX in Braf^{V600E} Cells Permits Tumor Control by Innate and Adaptive Immune Mechanisms

Given the COX-dependent phenotypes described above, we sought to establish the contribution of COX to the ability of Braf^{V600E} melanoma cells to grow in immunocompetent mice. Notably, *Ptgs1/Ptgs2*$^{-/-}$ cells formed spontaneously regressing tumors in WT mice in contrast to a COX-sufficient control clone which, despite having undergone the CRISPR/Cas9-mediated targeting procedure, retained COX-1 and COX-2 expression (Figure 1E) and grew similarly to the parental cells (Figure 4A). Importantly, *Ptgs1/Ptgs2*$^{-/-}$ tumors were able to grow in *Rag1*$^{-/-}$, *Tap1*$^{-/-}$, and *Batf3*$^{-/-}$ hosts (Figures 4A and 4B), indicating that prostanoid deficiency did not impair tumor formation in a cell-intrinsic fashion but rather acted to prevent CD8α^+/CD103$^+$ DC-dependent rejection mediated by CD8$^+$ T lymphocytes. Similar results were obtained using two other independent *Ptgs1/Ptgs2*$^{-/-}$ clones generated using a different set of single-guide RNAs (sgRNAs) (Figures 1E and S6A). Thus, COX activity is a key driver of adaptive immune escape by Braf^{V600E} melanoma cells.

Tumors formed by *Ptgs1/Ptgs2*$^{-/-}$ Braf^{V600E} cells were noticeably smaller than their COX-sufficient counterparts even before adaptive immunity is expected to impact on tumor size (Figure 4A). As none of the *Ptgs1/Ptgs2*$^{-/-}$ cell lines showed an obvious proliferative impairment in vitro (Figure S6B), we investigated whether a T and B lymphocyte-independent innate immune response was responsible. Given the COX-dependent inhibition of ISGs observed in the tumor microenvironment (see above), we examined the role of host type I IFN signaling in initial tumor growth control. We found that *Ptgs1/Ptgs2*$^{-/-}$ tumors grew considerably faster in *Ifnar1*$^{-/-}$ mice than in WT animals during the first 8 to 10 days post-inoculation, indistinguishably from tumors formed by parental COX-expressing Braf^{V600E} cells (Figure 4C). Of note, the growth of the latter was unaffected by IFNAR deficiency, consistent with the fact that they do not display an IFN signature (Figures 2B and 2D). These data indicate that an early type I IFN-dependent innate immune response restricts the growth of *Ptgs1/Ptgs2*$^{-/-}$ cells.

Finally, we assessed the development of immunity following challenge with *Ptgs1/Ptgs2*$^{-/-}$ tumors. Most mice that rejected *Ptgs1/Ptgs2*$^{-/-}$ cells were resistant to a subsequent challenge with unmodified parental Braf^{V600E} melanoma cells (Figure 4D), implying the development of immunity to shared target antigens and excluding a scenario of cancer immune privilege driven locally by tumor-derived prostanoids.

An Essential Role for PGE_2 in Braf^{V600E}-Melanoma Immune Escape

COX-1 and COX-2 are essential for the production of multiple prostanoids, and COX deficiency can shunt arachidonic acid into different metabolic pathways (Ricciotti and FitzGerald, 2011). To evaluate the specific contribution of PGE_2, we generated Braf^{V600E} melanoma cells genetically deficient in microsomal prostaglandin E synthase (mPGES)-1 and -2 (referred to as *Pges*$^{-/-}$ cells), two of the enzymes specifically required for the synthesis of PGE_2, but not other prostanoids. PGES-deficient melanoma cells phenocopied COX-deficient ones in that their CM lacked BMMC immunomodulatory activity (Figure 4E), and they were spontaneously rejected in immune competent recipients but grew progressively in T- and B-cell-deficient hosts (Figure 4F). Thus, these data indicate a major and non-redundant role for PGE_2 among prostanoids in the ability of Braf^{V600E} melanoma cells to avoid immune destruction.

COX-Dependent Immune Escape Is a Feature of Different Mouse Cancer Cells

To extend our findings, we examined the ability of COX to facilitate immune escape of other mouse cancers. A melanoma cell line driven by expression of Nras^{G12D} in melanocytes (Pedersen et al., 2013) also produced PGE_2 and formed tumors that were practically identical in Rag-sufficient and Rag-deficient mice (Figure 4G). In contrast, Nras^{G12D} melanoma cells rendered genetically deficient in COX-2 were spontaneously rejected in WT mice but grew like parental COX-2-competent tumors in *Rag1*$^{-/-}$ hosts (Figure 4G). Mice that rejected *Ptgs2*$^{-/-}$ tumors subsequently rejected parental COX-competent Nras^{G12D} melanoma

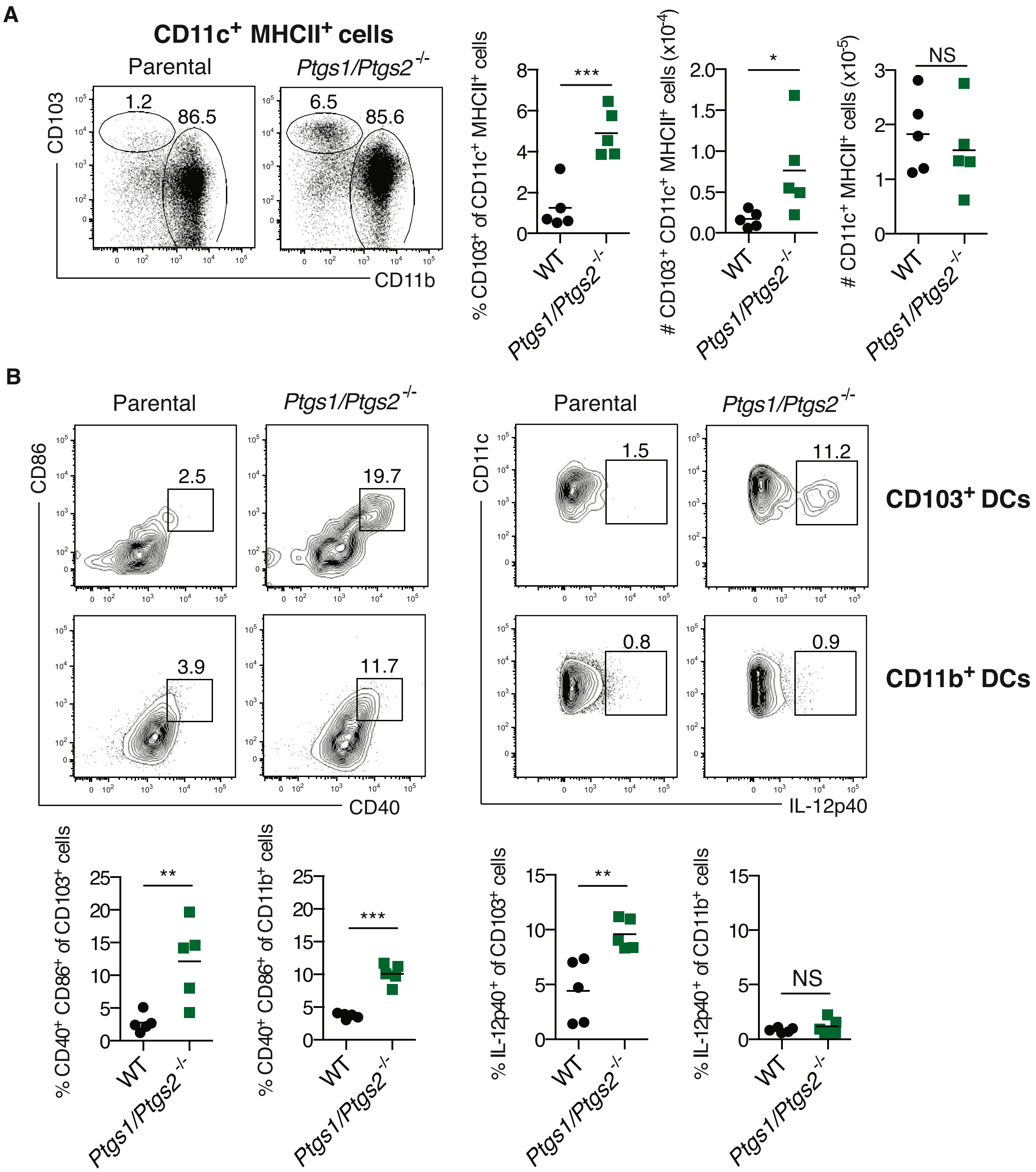

Figure 3. Tumor-Derived Prostanoids Prevent CD103$^+$ DC Accumulation and Activation

(A and B) WT mice were inoculated with 10^6 parental or *Ptgs1/Ptgs2*$^{-/-}$ Braf^{V600E} cells and tumor-infiltrating DCs were analyzed 4 days later. (A) Left: representative fluorescence-activated cell sorting (FACS) plots for CD103 versus CD11b within a CD11c$^+$ MHCII$^+$ DC gate. Right: percentage and number (#) of CD103$^+$ CD11c$^+$ MHCII$^+$ or CD11c$^+$ MHCII$^+$ cells. (B) Upper: representative FACS plots for CD11c versus IL-12p40 or CD86 versus CD40 within a CD103$^+$ or CD11b$^+$ CD11c$^+$ MHCII$^+$ gate. Lower: percentage of IL-12p40$^+$ or CD86$^+$ CD40$^+$ within CD103$^+$ or CD11b$^+$ CD11c$^+$ MHCII$^+$ cells. Each symbol in (A) and (B) represents an independent tumor. Samples were compared using two-tailed Student's t test. *$p < 0.05$, **$p < 0.01$, ***$p < 0.001$.

cells (Figure 4H), indicating the development of immunological memory and underscoring the presence of cryptic rejection antigens in parental Nras^{G12D} melanoma cells. In contrast to experiments with Braf^{V600E} cells, we did not find an obvious component of COX-dependent early innate immune control of Nras^{G12D} cells via type I IFN (data not shown).

To assess cancers other than melanoma and use mouse strains other than C57BL/6, we chose CT26 colorectal and 4T1 breast cancer cell lines that grow in BALB/c mice and also display constitutively active RAS/RAF/MEK/ERK signaling (Castle et al., 2014; Phan et al., 2013) and produce PGE_2 (Figures 5A and S4). Like the melanoma lines, these cancer cells exerted

immunomodulatory effects on BMMCs in vitro and grew identically in WT and T-cell-deficient nude mice (Figures 5A and 5B). In either case, genetic ablation of COX rendered the cells unable to produce PGE_2, abrogated their immunomodulatory effects on BMMCs, and allowed a marked degree of T-cell-dependent tumor growth control (Figures 5A and 5B). As for $Braf^{V600E}$ cells, COX-deficiency was associated with a shift in the inflammatory profile at the tumor site, with reduced expression of tumor promoting factors, such as *Il6* or *Il1b* and increased levels of mediators associated with anti-tumor immune pathways (Figure 5C). Finally, mice that fully rejected COX-deficient CT26 or 4T1 tumors were immune to subsequent challenge with the respective COX-competent parental lines (data not shown). We conclude that prostanoid-dependent subversion of the inflammatory response and escape from anti-cancer immunity is a general feature of COX-expressing tumors.

COX Inhibitors Enhance the Efficacy of Immunotherapy with an Anti-PD-1 Blocking Antibody

Aspirin blocks both COX-1 and COX-2 and can be administered to mice in drinking water. However, this had no effect on the progression of implanted COX-competent $Braf^{V600E}$ melanoma cells (Figures 6A–6C), perhaps because of incomplete inhibition of COX activity. However, even a modest degree of COX inhibition might help enhance the efficacy of immunotherapies, including those based on immune checkpoint blockade. Consistent with that notion, aspirin in the drinking water, in combination with treatment with anti-PD-1 monoclonal antibody, promoted much more rapid tumor regression and eradication of $Braf^{V600E}$ melanoma cells than anti-PD-1 alone (Figures 6A and 6B). The potent synergy of the aspirin/anti-PD-1 combination was fully dependent on adaptive immunity as it was lost in $Rag1^{-/-}$ mice (Figure 6B). It was also manifest in experiments using a larger inoculum of tumor cells, in which anti-PD-1 blockade alone had no effect (Figure 6C). Mice that fully eradicated COX-sufficient tumors upon treatment with aspirin + anti-PD-1 were immune to a subsequent challenge in the absence of further treatment (Figure 6D). Administration of celecoxib, a COX-2-specific inhibitor, also significantly synergized with anti-PD-1 treatment (Figure 6E), albeit to a lesser degree than aspirin, possibly due to suboptimal COX-2 inhibition and/or a potential contribution of COX-1-derived PGE_2. Finally, we addressed whether the synergy of the combination could be observed with tumors besides melanoma. Notably, treatment of mice bearing CT26 colorectal tumors with aspirin and anti-PD-1-induced tumor growth control and rapid and complete shrinkage in 30% of mice, whereas monotherapy showed little efficacy (Figure 6F). These experiments suggest that COX inhibitors could be useful additions to immune checkpoint blockade or conventional treatment of cancer patients so long as prostanoids also constitute a means of tumor immune escape in humans.

The COX-Dependent Inflammatory Signature Is Conserved in Human Cutaneous Melanoma Biopsies

To evaluate the latter, we asked whether evidence for COX-dependent immune modulation can be found in human melanomas. We correlated PTGS2 (encoding COX-2) mRNA expression levels in human melanoma biopsies containing tumor, as well as stromal and infiltrating, cells with levels of mRNAs encoding various immune mediators, including those that we found to be controlled by COX-2 in the mouse models. Strikingly, mRNA expression levels for IL-6, G-CSF, CXCL1, and other known tumor-promoting inflammatory factors showed strong positive correlation with those of PTGS2 in samples from human cutaneous melanoma (Figures 7A and 7C). No correlation was observed between PTGS2 levels and levels of markers indicative of total leukocyte (CD45), regulatory T cell (FOXP3), or B cell (CD19 and CD20) presence (Figure 7B). In contrast, PTGS2 mRNA levels were inversely correlated with CD8A and CD8B transcript levels, a measure of the presence of $CD8^+$ T cells in tumors associated with longer survival and favorable treatment outcome (Fridman et al., 2012; Gajewski et al., 2010). Similarly, expression of PTGS2 was inversely correlated with that of CXCL10 and CXCL9, chemokines associated with cytotoxic T cell recruitment (Fridman et al., 2012; Gajewski et al., 2010) (Figures 7A and 7D). We also found a significant and consistent negative association between PTGS2 expression and that of numerous ISGs (Figures 7A and 7E). Together, these data indicate a qualitative change in immune infiltrate composition that is driven by COX expression and shows remarkable parallels between mice and human.

DISCUSSION

The extent to which the immune system acts as a natural barrier to tumor progression has been the subject of long-standing debate (Hanahan and Weinberg, 2011). Data from both mouse and human cancers over the last two decades has lent support to the notion that neoplastic development is associated with an immunoediting process, whereby the immune system selects the outgrowth of less immunogenic tumor cells (Rooney et al., 2015; Schreiber et al., 2011; Vesely et al., 2011). The mechanisms underlying immunoediting are only beginning to be explored and include selection for tumor cells that lose dominant tumor rejection antigens (DuPage et al., 2012; Matsushita et al., 2012). Here, we uncover PGE_2-dependent suppression of myeloid cell activation as a potent additional mechanism of tumor immune escape. Our findings suggest that immunoediting can result in selection of tumors producing immunosuppressive factors, which block initial type I IFN-dependent innate immune cell activation and/or prevent subsequent T cell activity against tumor antigens. The latter indicates that edited tumor cells may continue to express relevant target antigens that can be functionally unmasked upon removal of tumor-derived suppressive factors that subvert myeloid cell, including DC, function. These findings have obvious therapeutic implications, as discussed below. In addition, they help to explain apparently contradictory earlier findings suggesting that genetically driven mouse cancers are not subject to immune surveillance (Willimsky and Blankenstein, 2005) by showing that production of suppressive factors by the tumor is in fact a feature of immunoediting. The low immunogenicity of tumors can therefore result from immune sculpting of their antigenic and/or immunostimulatory properties.

Our experiments rely partly on the ability to genetically engineer cancer cells using CRISPR/Cas9-mediated technology to assess the contribution of specific tumor pathways to immune

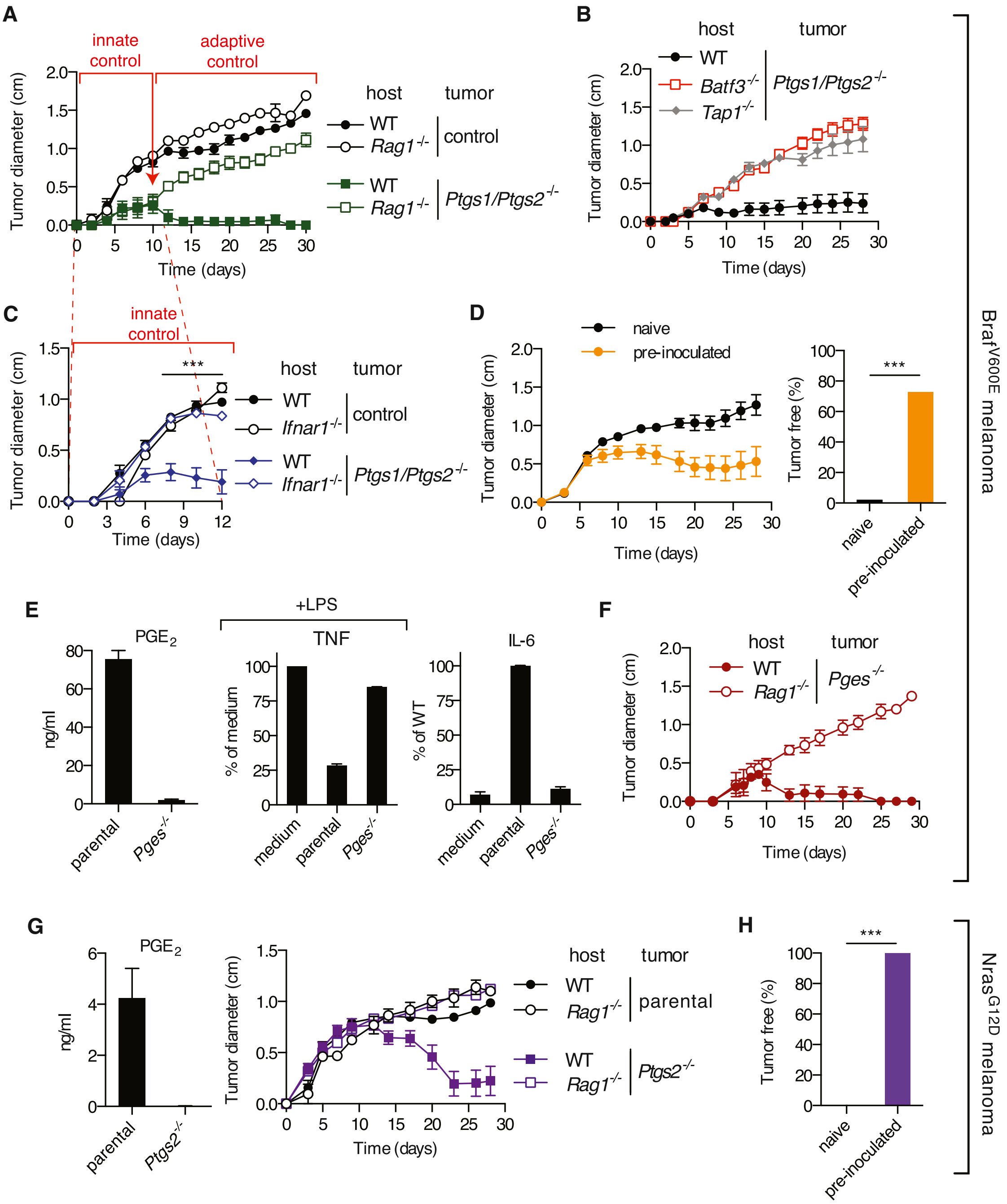

Figure 4. Genetic Ablation of COX in Braf^{V600E} or Nras^{G12D} Melanoma Cells Enables Immune-Dependent Tumor Eradication

(A–C) Growth of tumors formed following implantation of 10^5 control or *Ptgs1/Ptgs2*$^{-/-}$ Braf^{V600E} cells into WT C57BL/6 (A–C), *Rag1*$^{-/-}$ (A), *Batf3*$^{-/-}$, *Tap1*$^{-/-}$ (B), or *Ifnar1*$^{-/-}$ (C) mice.

(D) Growth of parental Braf^{V600E} cells following implantation into naive WT C57BL/6 mice or mice that previously rejected *Ptgs1/Ptgs2*$^{-/-}$ Braf^{V600E} tumors (pre-inoculated). Data are compiled from three independent experiments and presented as tumor growth profile (left) and as percentage of tumor-free mice at 6 weeks post-parental tumor inoculation (right).

(E) Concentration of PGE$_2$ in CM from confluent parental or *Pges*$^{-/-}$ cell cultures cells or of TNF and IL-6 in the supernatant of an overnight culture of BMMCs cultured as in Figure 1 in presence of CM from the indicated cell line and expressed as in Figure 1G.

(F) Growth profile of tumors formed following implantation of 10^5 parental or *Pges*$^{-/-}$ Braf^{V600E} cells into WT or *Rag1*$^{-/-}$ mice.

(G) Concentration of PGE$_2$ in CM from confluent cell cultures or growth profile of tumors formed following implantation of 10^5 parental or *Ptgs2*$^{-/-}$ Nras^{G12D} cells into WT or *Rag1*$^{-/-}$ mice (right). (H) The percentage of tumor-free mice at 6 weeks post-implantation of parental Nras^{G12D} cells into naive WT C57BL/6 mice or

(legend continued on next page)

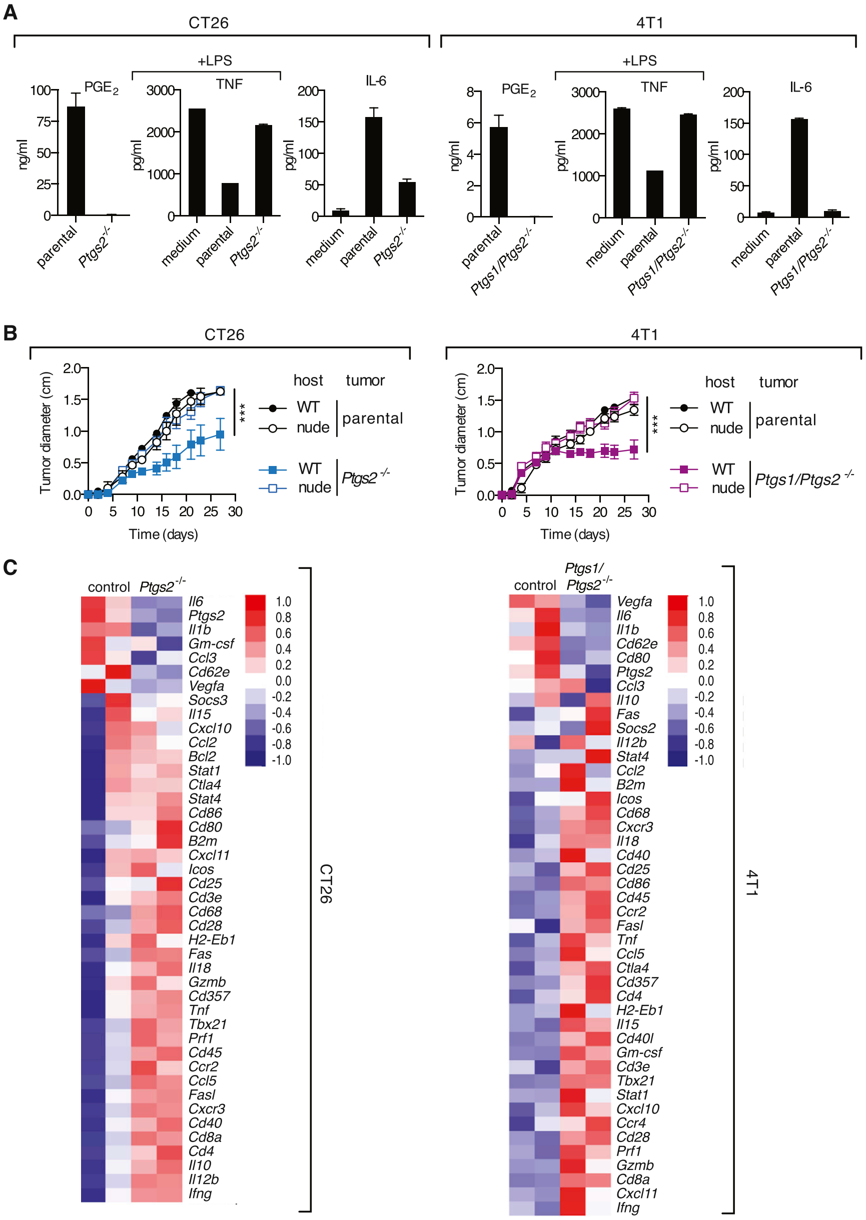

Figure 5. COX Ablation in Colorectal or Breast Cancer Cells Promotes Cancer-Inhibiting Inflammation and T-Cell-Dependent Tumor Growth Control

(A) Concentration of PGE_2 in CM from confluent parental and COX-deficient CT26 and 4T1 cell cultures cells and of TNF and IL-6 in the supernatant of an overnight culture of BMMCs cultured as in Figure 1 in presence of the indicated cell line CM.

(B) Growth profile of tumors formed following implantation of 10^5 parental or *Ptgs2*$^{-/-}$ CT26 colorectal or of parental or *Ptgs1/Ptgs2*$^{-/-}$ 4T1 breast cancer cells into WT Balb/c or nude mice. Data are presented as average tumor diameters ± SEM and are representative of at least three independent experiments with four to six mice per group. Tumor growth profiles were compared using two-way ANOVA. ***$p < 0.001$.

(C) WT Balb/c mice were inoculated with 10^6 parental, *Ptgs2*$^{-/-}$ CT26 or *Ptgs1/Ptgs2*$^{-/-}$ 4T1 cells, and 4 days later the expression of an array of immune-associated genes was determined by qPCR in whole-tumor homogenates. Heatmaps for a selected list of genes show log2 ΔCT values normalized to *hprt* of two biological replicates for each value. The genes are ordered from highest to lowest by fold change in parental relative to *Ptgs2*$^{-/-}$ or *Ptgs1/Ptgs2*$^{-/-}$ samples.

escape in the absence of alterations in the same pathways in host stroma. Off-target Cas9 nuclease activity can be a concern (e.g., Frock et al., 2015), but because we have used multiple sgRNAs to ablate *Ptges1*, *Ptges2*, and/or *Pges1/2* in four different cancer cell lines that, in all cases, become susceptible to immune-mediated control, we believe that on-target ablation of the ability of the tumor cells to produce PGE_2 accounts for our findings. Similarly, although the introduction of nucleotide insertions and/or deletions is inherent to the CRISPR engineering process, it is extremely improbable that they would in all cases inadvertently lead to the generation of neo-self determinants that can be efficiently processed and presented on H-2 MHC class I molecules for recognition by $CD8^+$ T cells. We therefore believe that the antigen targets of $CD8^+$ T cells in COX-deficient tumors are cancer-associated antigens, mutated proteins, and/or minor histocompatibility antigens that are shared with the parental tumor as denoted by the fact that immunity to the latter develops in mice that reject the former. This finding, in turn, argues against the possibility that tumor-derived prostanoids promote immune evasion merely by preventing tumor infiltration by lymphocytes or access of the latter to their targets (Joyce and Fearon, 2015). In such a scenario, COX-competent parental cells should create an immune-privileged site and form progressive tumors even when the host has previously rejected COX-deficient cells.

Antigen-presenting cells, in particular DCs, are greatly affected by tumor-derived PGE_2 and likely to be an important target of the lipid for tumor immune escape. While DCs have

mice that previously rejected *Ptgs2*$^{-/-}$ $Nras^{G12D}$ tumors (pre-inoculated). All growth profiles are presented as average tumor diameters ± SEM and are representative of at least two independent experiments with four to six mice per group. Tumor growth profiles were compared using two-way ANOVA and the percentage of tumor-free mice using Fisher's exact test. ***$p < 0.001$.
See also Figure S6.

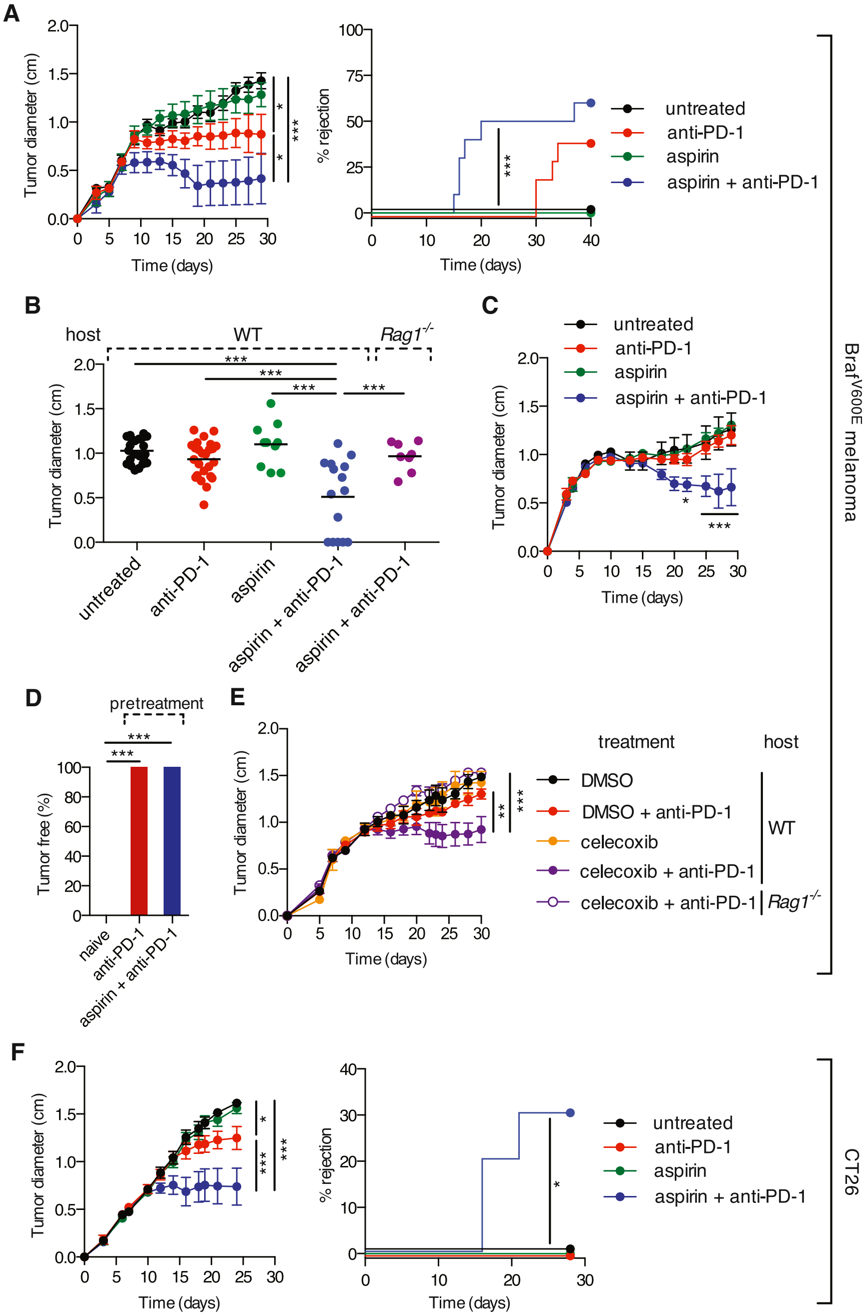

Figure 6. COX Inhibition Synergizes with Anti-PD-1 Blockade in Immune-Dependent Tumor Growth Control

(A) Left: growth of parental COX-competent tumors following implantation of 10^5 $Braf^{V600E}$ melanoma cells into C57BL/6 mice. Mice received aspirin in the drinking water and/or 200 μg of anti-PD-1 monoclonal antibody i.p. every 3–4 days from day 3 to day 24. Right: the percentage of mice that fully rejected tumors over time is shown.

(B) Pooled tumor diameters at 19 days post-implantation of $Braf^{V600E}$ melanoma cells into WT or $Rag1^{-/-}$ mice treated as in (A). Each dot represents one independent tumor.

(C) As in (A) but using an inoculum of 10^6 melanoma cells.

(D) The percentage of tumor-free mice at 6 weeks post-implantation of parental $Braf^{V600E}$ cells into C57BL/6 mice that were untreated (n = 15) (naive) or that previously rejected $Braf^{V600E}$ cells following anti-PD-1 (n = 6) or aspirin + anti-PD-1 treatment (n = 8) (pre-treatment).

(E) As in (A) but C57BL/6 mice received celecoxib i.p. daily from day 0.

(F) As in (A) but Balb/c mice received 10^5 CT26 colorectal cells. Growth profiles are presented as average tumor diameters ± SEM and are representative of at least two independent experiments with five mice per group.

Samples were compared using two-way ANOVA (A, C, E, and F), one-way ANOVA (B), Fisher's exact test (D), and log rank test (A and F). $^{*}p < 0.05$, $^{**}p < 0.01$, $^{***}p < 0.001$.

been most prominently studied for their ability to prime anti-tumor T cells in lymph nodes, they are also emerging as key players at the tumor site. Recent studies have indicated that tumor accumulation of rare Batf3-dependent DCs bearing the CD103 marker is associated with good prognosis and immune-mediated control across mouse and human species (Broz et al., 2014; Ruffell et al., 2014). Batf3-dependent DCs appear to act by restimulating cytotoxic T lymphocyte (CTL) at the tumor site, in part by locally providing IL-12. Their dual activity in cross-priming anti-tumor CTL within lymph nodes and restimulating CTL within tumors probably contributes to the general Batf3-dependence of anti-tumor immunity that has been observed in mice (Diamond et al., 2011; Fuertes et al., 2011; Hildner et al., 2008) and to the suppression of anti-tumor immunity by PGE_2 that we observe here. However, it is likely that PGE_2 acts on additional cell types, including CTLs themselves. Indeed, CTL survival and function has been recently shown to be directly impaired by PGE_2 in the context of chronic viral infection (Chen et al., 2015). Furthermore, we find that COX activity prevents activation of the type I IFN system in the tumor microenvironment, in agreement with recent reports demonstrating an inhibitory role for PGE_2 on type I IFN production during infection with influenza or mycobacteria (Coulombe et al., 2014; Mayer-Barber et al., 2014). This may be especially relevant to melanoma where the expression of a type I IFN signature associates with spontaneous remissions (Wenzel et al., 2005) and increased relapse-free survival (Bald et al., 2014). Finally, tumor-derived prostanoids directly induce the production by myeloid cells of known cancer-promoting factors such as IL-6, CXCL1, and G-CSF, effectively shifting the tumor microenvironment from one favorable to tumor eradication to one that has pro-tumor activity.

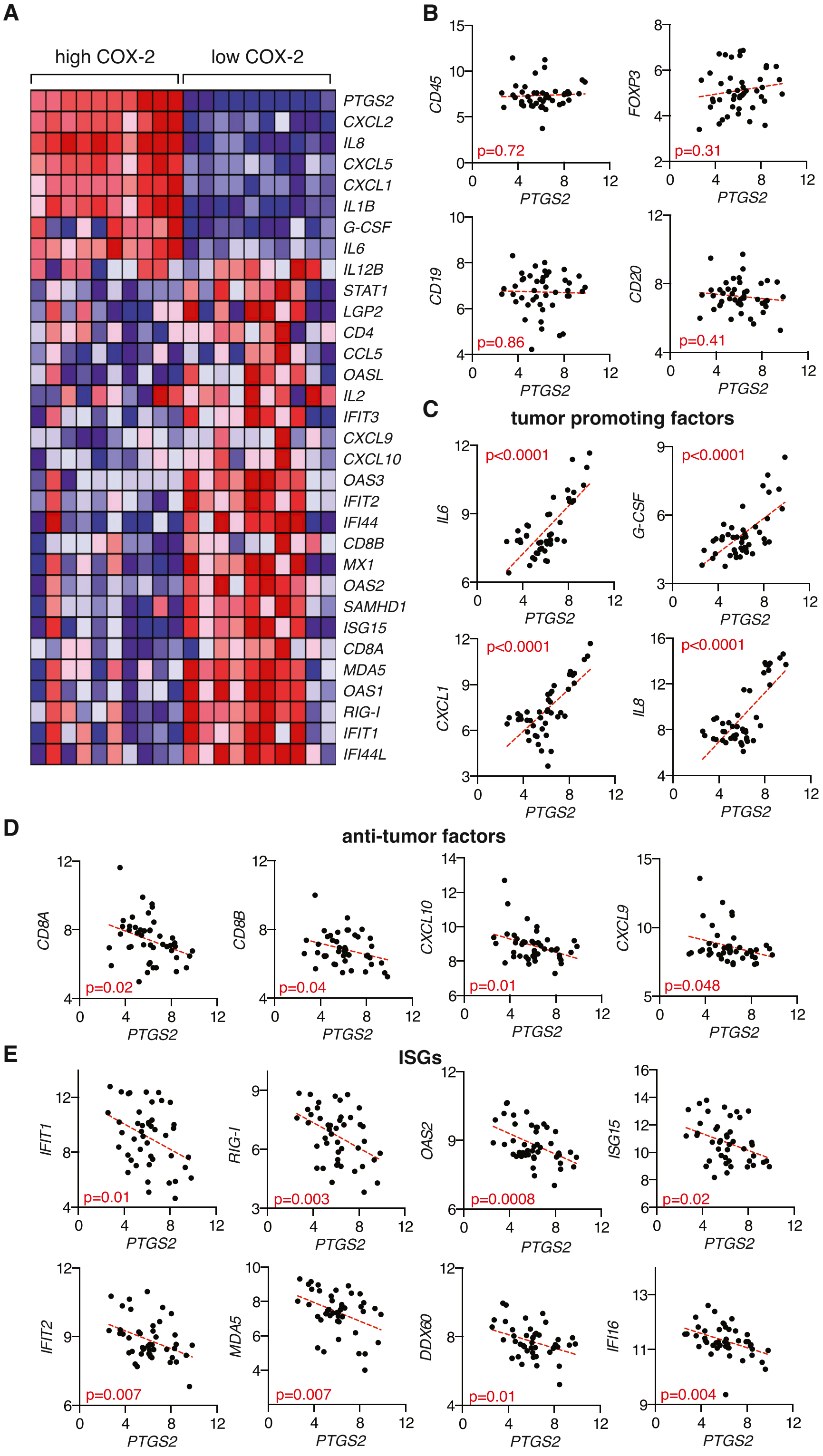

Figure 7. COX-2 Levels in Human Melanoma Biopsies Correlate Positively with Tumor-Promoting Factors and Negatively with Factors Associated with CTL Infiltration and Type I IFN Signaling

(A–E) Microarray expression data (Talantov et al., 2005) from human cutaneous melanoma biopsies containing tumor cells, stroma, and infiltrate were analyzed for the association of *PTGS2* expression with that of several immune-related genes.

(A) Heatmap for a selected list of genes showing log2 expression signal for 20% of samples with highest (high COX-2) and lowest (low COX-2) *PTGS2* expression. Genes were clustered using a Euclidean distance matrix and average linkage clustering. Red indicates higher expression, and blue indicates low expression relative to the mean expression of the gene across all samples.

(B) Correlation data for *PTGS2* versus *CD45 (PTPRC)*, *FOXP3*, *CD19*, and *CD20* expression.

(C) Correlation data for *PTGS2* versus *IL-6*, *G-CSF (CSF3)*, *CXCL1*, and *IL-8* expression.

(D) Correlation data for *PTGS2* versus *CD8A*, *CD8B*, *CXCL10*, and *CXCL9*.

(E) Correlation data for *PTGS2* versus the following ISGs: *IFIT1*, *IFIT2*, *RIG-I (DDX58)*, *MDA5 (IFIH1)*, *OAS2*, *DDX60*, *ISG15*, and *IFI16*.

In (B)–(E), all cutaneous melanoma samples (n = 45) from the dataset (Talantov et al., 2005) were included in the analysis, with each dot representing one sample. The statistical significance of the correlation was determined using the Pearson's correlation coefficient. A linear regression-fitting curve is shown as a dotted red line.

Interestingly, COX-2 is often overexpressed in several human cancers, including colorectal, breast, stomach, lung, and pancreatic tumors (Dannenberg and Subbaramaiah, 2003). Whether melanomas similarly express abnormal levels of COX-2 remains unclear (Becker et al., 2009; Denkert et al., 2001; Goulet et al., 2003; Kuźbicki et al., 2006). Our analysis of a publically available dataset of a cohort of human melanoma biopsies (Talantov et al., 2005) suggests that the natural variability in COX-2 expression levels within samples might be of functional relevance. Thus, samples with high COX-2 levels showed higher expression of numerous tumor-promoting factors, including those whose expression was directly controlled by COX in the mouse model. Likewise, COX-2^{low} melanomas showed a qualitative change in infiltrate composition, displaying increased expression of anti-tumor mediators and hallmarks of activation of the type I IFN system. Thus, our combined analysis of the mouse model and the human samples argues for COX activity as a common mechanism co-opted by cancer cells to promote immune escape across species.

Prostanoids have been implicated in carcinogenesis through enhancement of cancer cell survival, proliferation, invasion,

and angiogenesis (Wang and Dubois, 2010). COX inhibitors were recently reported to enhance the efficacy of antiangiogenic therapy in pre-clinical models by inhibiting VEGF-independent PGE_2-induced tumor angiogenesis (Xu et al., 2014). As such, it is remarkable that COX-deficient cancer cells are able to grow indistinguishably from their COX-competent counterparts in immunodeficient mice. These results indicate that, at least for some tumors, the main role of cancer-cell-derived prostanoid production is to promote immune evasion and that any effects on angiogenesis or tumor cell survival and proliferation are likely secondary to immune suppression or redundant with stroma-derived PGE_2. Notably, our findings that COX-2 expression depends on active RAF/MEK signaling suggests that reduced production of PGE_2 by melanoma cells may contribute to the immune-dependent anti-cancer activity elicited by BRAF inhibitors (Frederick et al., 2013; Knight et al., 2013). Nevertheless, COX-2 upregulation is likely to also be driven by MAPK-independent pathways, consistent with the presence of multiple regulatory elements in the *PTGS2* promoter.

We find a remarkable conservation between signatures of COX-dependent subversion of inflammation across mouse and human melanoma. We therefore propose that COX-2 levels and COX-dependent inflammatory mediators in human melanoma and other cancers might constitute useful biomarkers predictive of prognosis and treatment outcome, including in response to checkpoint blockade inhibitors, such as anti-CTLA4 and anti-PD-1/PD-L1. Finally, our data show that COX inhibitors act synergistically with anti-PD-1 mAb in pre-clinical models. We therefore speculate that COX inhibitors, reported to reduce the risk of several cancers, including colorectal (Rothwell et al., 2010), gastric (Tian et al., 2010), breast cancer (Gierach et al., 2008), and, even, melanoma (Gamba et al., 2013), might help unleash anti-cancer immunity and thereby constitute useful additions to the arsenal of conventional and immune-based cancer therapies, most notably those based on immune checkpoint blockade.

EXPERIMENTAL PROCEDURES

Mice

All animal experiments were performed in accordance with national and institutional guidelines for animal care and were approved by an institutional Animal Ethics Committee and by the Home Office, UK.

Cancer Cell Lines

Cells were cultured under standard conditions and were confirmed to be mycoplasma free. $Braf^{V600E}$ and $Nras^{G12D}$ melanoma cell lines were established from C57BL/6 $Braf^{+/LSL-V600E}$;Tyr::$CreERT2^{+/o}$;$p16^{INK4a-/-}$ (Dhomen et al., 2009) and C57BL/6 $Nras^{+/LSL-G12D}$;Tyr::$CreERT2^{+/o}$ (Pedersen et al., 2013) mice, respectively. CT26, 4T1, and EL4 cells were from ATCC. $Ptgs2^{-/-}$, *Ptgs1*/$Ptgs2^{-/-}$, and $Pges^{-/-}$ cells were generated by CRISPR/Cas9-mediated genome engineering using the CRISPR design tool provided by the Zhang lab (http://www.genome-engineering.org). Correctly targeted clones were selected based on their inability to produce PGE_2, and genetic ablation of COX-1, COX-2, mPGES-1, and mPGES-2 was verified by sequencing.

Tumor Cell Injections

Tumor cells were harvested by trypsinization, washed three times with PBS, and injected subcutaneously into the right flank of recipient mice at 10^5 to 10^6 cells in 100 μl of endotoxin-free PBS. Tumor cells were >98% viable at the time of injection as determined by propidium iodide staining. Tumor size was quantified as the mean of the longest diameter and its perpendicular. For COX inhibition in vivo, aspirin was administered in the drinking water at 600 μg/ml 1 to 3 days before injection of tumor cells and replaced every 3 days. Alternatively, mice received 200 μl of celecoxib (Sigma and LC Laboratories) intraperitoneally (i.p.) at 500 μg/ml (12.5% DMSO in PBS) daily from day 0. Anti-PD-1 monoclonal antibody (clone RMP1-14, BioXCell) was administered i.p. at 200 μg/mouse from day 3 post-tumor cell inoculation every 3 to 4 days for a maximum of six injections.

In Vitro Culture

Mouse BMMCs were generated using GM-CSF as described (Helft et al., 2015). Cells were plated at 0.5- to 1 × 10^6 cells/ml in 96-well plates at 37°C in absence or presence of 100 μl of conditioned medium from tumor cells plus or minus LPS (10 to 100 ng/ml) in a total volume of 200 μl. After overnight culture, cytokine and chemokine concentration in the supernatant was determined by ELISA or by cytometric bead array using standard procedures.

qPCR

Tumors were collected and homogenized, and total RNA was isolated with Trizol reagent (Invitrogen) and further purified on RNAeasy columns (QIAGEN). cDNA was synthesized using SuperscriptII reverse transcriptase (Invitrogen). Expression of an array of immune genes was performed using a TaqMan mouse immune array (v.2.1), following the manufacturer's instructions.

Human Microarray Dataset Analysis

Raw CEL files from the microarray dataset GSE3189 were downloaded from GEO (http://www.ncbi.nlm.nih.gov).

Statistics

Statistical significance was determined using an unpaired two-tailed Student's t test, one-way ANOVA, two-way ANOVA, Fisher's exact test, log rank test, and the Pearson correlation coefficient as indicated. A p value < 0.05 was considered significant (* p < 0.05; ** p < 0.01; *** p < 0.001).

SUPPLEMENTAL INFORMATION

Supplemental Information includes Supplemental Experimental Procedures and six figures and can be found with this article online at http://dx.doi.org/10.1016/j.cell.2015.08.015.

AUTHOR CONTRIBUTIONS

S.Z. conducted experiments with assistance from A.G.V., J.P.B, K.J.S., and S.E.A. P.C. and S.Z. carried out bioinformatic analysis. A.G.V. and S.Z. carried out cell engineering. N.R. managed mouse stocks. M.R.G. and R.M. provided key reagents. A.G.V., S.A.Q., and E.S. provided advice, analyzed data, and contributed to experimental design. S.Z. and C.R.S. designed the study, analyzed data, and wrote the manuscript.

ACKNOWLEDGMENTS

This work was funded by Cancer Research UK, The Francis Crick Institute, and a European Research Council Advanced Researcher Grant. A.G.V. was supported by an EMBO Long-Term Fellowship and a Rubicon Fellowship from the Netherlands Organization for Scientific Research. The authors are grateful to members of the Immunobiology Laboratory for assistance and helpful discussions.

Received: October 28, 2014
Revised: April 22, 2015
Accepted: July 16, 2015
Published: September 3, 2015

REFERENCES

Bald, T., Landsberg, J., Lopez-Ramos, D., Renn, M., Glodde, N., Jansen, P., Gaffal, E., Steitz, J., Tolba, R., Kalinke, U., et al. (2014). Immune cell-poor

melanomas benefit from PD-1 blockade after targeted type I IFN activation. Cancer Discov. *4*, 674–687.

Balkwill, F., Charles, K.A., and Mantovani, A. (2005). Smoldering and polarized inflammation in the initiation and promotion of malignant disease. Cancer Cell *7*, 211–217.

Becker, M.R., Siegelin, M.D., Rompel, R., Enk, A.H., and Gaiser, T. (2009). COX-2 expression in malignant melanoma: a novel prognostic marker? Melanoma Res. *19*, 8–16.

Broz, M.L., Binnewies, M., Boldajipour, B., Nelson, A.E., Pollack, J.L., Erle, D.J., Barczak, A., Rosenblum, M.D., Daud, A., Barber, D.L., et al. (2014). Dissecting the tumor myeloid compartment reveals rare activating antigen-presenting cells critical for T cell immunity. Cancer Cell *26*, 638–652.

Castle, J.C., Loewer, M., Boegel, S., de Graaf, J., Bender, C., Tadmor, A.D., Boisguerin, V., Bukur, T., Sorn, P., Paret, C., et al. (2014). Immunomic, genomic and transcriptomic characterization of CT26 colorectal carcinoma. BMC Genomics *15*, 190.

Chen, J.H., Perry, C.J., Tsui, Y.-C., Staron, M.M., Parish, I.A., Dominguez, C.X., Rosenberg, D.W., and Kaech, S.M. (2015). Prostaglandin E2 and programmed cell death 1 signaling coordinately impair CTL function and survival during chronic viral infection. Nat. Med. *21*, 327–334.

Coulombe, F., Jaworska, J., Verway, M., Tzelepis, F., Massoud, A., Gillard, J., Wong, G., Kobinger, G., Xing, Z., Couture, C., et al. (2014). Targeted prostaglandin E2 inhibition enhances antiviral immunity through induction of type I interferon and apoptosis in macrophages. Immunity *40*, 554–568.

Coussens, L.M., Zitvogel, L., and Palucka, A.K. (2013). Neutralizing tumor-promoting chronic inflammation: a magic bullet? Science *339*, 286–291.

Dannenberg, A.J., and Subbaramaiah, K. (2003). Targeting cyclooxygenase-2 in human neoplasia: rationale and promise. Cancer Cell *4*, 431–436.

Denkert, C., Köbel, M., Berger, S., Siegert, A., Leclere, A., Trefzer, U., and Hauptmann, S. (2001). Expression of cyclooxygenase 2 in human malignant melanoma. Cancer Res. *61*, 303–308.

Dhomen, N., Reis-Filho, J.S., da Rocha Dias, S., Hayward, R., Savage, K., Delmas, V., Larue, L., Pritchard, C., and Marais, R. (2009). Oncogenic Braf induces melanocyte senescence and melanoma in mice. Cancer Cell *15*, 294–303.

Diamond, M.S., Kinder, M., Matsushita, H., Mashayekhi, M., Dunn, G.P., Archambault, J.M., Lee, H., Arthur, C.D., White, J.M., Kalinke, U., et al. (2011). Type I interferon is selectively required by dendritic cells for immune rejection of tumors. J. Exp. Med. *208*, 1989–2003.

Dunn, G.P., Bruce, A.T., Sheehan, K.C.F., Shankaran, V., Uppaluri, R., Bui, J.D., Diamond, M.S., Koebel, C.M., Arthur, C., White, J.M., and Schreiber, R.D. (2005). A critical function for type I interferons in cancer immunoediting. Nat. Immunol. *6*, 722–729.

DuPage, M., Mazumdar, C., Schmidt, L.M., Cheung, A.F., and Jacks, T. (2012). Expression of tumour-specific antigens underlies cancer immunoediting. Nature *482*, 405–409.

Frederick, D.T., Piris, A., Cogdill, A.P., Cooper, Z.A., Lezcano, C., Ferrone, C.R., Mitra, D., Boni, A., Newton, L.P., Liu, C., et al. (2013). BRAF inhibition is associated with enhanced melanoma antigen expression and a more favorable tumor microenvironment in patients with metastatic melanoma. Clin. Cancer Res. *19*, 1225–1231.

Fridman, W.-H., Pagès, F., Sautès-Fridman, C., and Galon, J. (2012). The immune contexture in human tumours: impact on clinical outcome. Nat. Rev. Cancer *12*, 298–306.

Frock, R.L., Hu, J., Meyers, R.M., Ho, Y.-J., Kii, E., and Alt, F.W. (2015). Genome-wide detection of DNA double-stranded breaks induced by engineered nucleases. Nat. Biotechnol. *33*, 179–186.

Fuertes, M.B., Kacha, A.K., Kline, J., Woo, S.-R., Kranz, D.M., Murphy, K.M., and Gajewski, T.F. (2011). Host type I IFN signals are required for antitumor CD8+ T cell responses through CD8alpha+ dendritic cells. J. Exp. Med. *208*, 2005–2016.

Gajewski, T.F., Louahed, J., and Brichard, V.G. (2010). Gene signature in melanoma associated with clinical activity: a potential clue to unlock cancer immunotherapy. Cancer J. *16*, 399–403.

Gajewski, T.F., Schreiber, H., and Fu, Y.-X. (2013). Innate and adaptive immune cells in the tumor microenvironment. Nat. Immunol. *14*, 1014–1022.

Gamba, C.A., Swetter, S.M., Stefanick, M.L., Kubo, J., Desai, M., Spaunhurst, K.M., Sinha, A.A., Asgari, M.M., Sturgeon, S., and Tang, J.Y. (2013). Aspirin is associated with lower melanoma risk among postmenopausal Caucasian women: the Women's Health Initiative. Cancer *119*, 1562–1569.

Gierach, G.L., Lacey, J.V., Jr., Schatzkin, A., Leitzmann, M.F., Richesson, D., Hollenbeck, A.R., and Brinton, L.A. (2008). Nonsteroidal anti-inflammatory drugs and breast cancer risk in the National Institutes of Health-AARP Diet and Health Study. Breast Cancer Res. *10*, R38.

Goulet, A.-C., Einsphar, J.G., Alberts, D.S., Beas, A., Burk, C., Bhattacharyya, A., Bangert, J., Harmon, J.M., Fujiwara, H., Koki, A., and Nelson, M.A. (2003). Analysis of cyclooxygenase 2 (COX-2) expression during malignant melanoma progression. Cancer Biol. Ther. *2*, 713–718.

Grivennikov, S.I., Greten, F.R., and Karin, M. (2010). Immunity, inflammation, and cancer. Cell *140*, 883–899.

Hanahan, D., and Weinberg, R.A. (2011). Hallmarks of cancer: the next generation. Cell *144*, 646–674.

Helft, J., Böttcher, J., Chakravarty, P., Zelenay, S., Huotari, J., Schraml, B.U., Goubau, D., and Reis e Sousa, C. (2015). GM-CSF mouse bone marrow cultures comprise a heterogeneous population of CD11c(+)MHCII(+) macrophages and dendritic cells. Immunity *42*, 1197–1211.

Hildner, K., Edelson, B.T., Purtha, W.E., Diamond, M., Matsushita, H., Kohyama, M., Calderon, B., Schraml, B.U., Unanue, E.R., Diamond, M.S., et al. (2008). Batf3 deficiency reveals a critical role for CD8alpha+ dendritic cells in cytotoxic T cell immunity. Science *322*, 1097–1100.

Joyce, J.A., and Fearon, D.T. (2015). T cell exclusion, immune privilege, and the tumor microenvironment. Science *348*, 74–80.

Kalinski, P. (2012). Regulation of immune responses by prostaglandin E2. J. Immunol. *188*, 21–28.

Knight, D.A., Ngiow, S.F., Li, M., Parmenter, T., Mok, S., Cass, A., Haynes, N.M., Kinross, K., Yagita, H., Koya, R.C., et al. (2013). Host immunity contributes to the anti-melanoma activity of BRAF inhibitors. J. Clin. Invest. *123*, 1371–1381.

Kuźbicki, L., Sarnecka, A., and Chwirot, B.W. (2006). Expression of cyclooxygenase-2 in benign naevi and during human cutaneous melanoma progression. Melanoma Res. *16*, 29–36.

Mantovani, A., Allavena, P., Sica, A., and Balkwill, F. (2008). Cancer-related inflammation. Nature *454*, 436–444.

Matsushita, H., Vesely, M.D., Koboldt, D.C., Rickert, C.G., Uppaluri, R., Magrini, V.J., Arthur, C.D., White, J.M., Chen, Y.-S., Shea, L.K., et al. (2012). Cancer exome analysis reveals a T-cell-dependent mechanism of cancer immunoediting. Nature *482*, 400–404.

Mayer-Barber, K.D., Andrade, B.B., Oland, S.D., Amaral, E.P., Barber, D.L., Gonzales, J., Derrick, S.C., Shi, R., Kumar, N.P., Wei, W., et al. (2014). Host-directed therapy of tuberculosis based on interleukin-1 and type I interferon crosstalk. Nature *511*, 99–103.

Page, D.B., Postow, M.A., Callahan, M.K., Allison, J.P., and Wolchok, J.D. (2014). Immune modulation in cancer with antibodies. Annu. Rev. Med. *65*, 185–202.

Pedersen, M., Küsters-Vandevelde, H.V.N., Viros, A., Groenen, P.J.T.A., Sanchez-Laorden, B., Gilhuis, J.H., van Engen-van Grunsven, I.A., Renier, W., Schieving, J., Niculescu-Duvaz, I., et al. (2013). Primary melanoma of the CNS in children is driven by congenital expression of oncogenic NRAS in melanocytes. Cancer Discov. *3*, 458–469.

Phan, V.T., Wu, X., Cheng, J.H., Sheng, R.X., Chung, A.S., Zhuang, G., Tran, C., Song, Q., Kowanetz, M., Sambrone, A., et al. (2013). Oncogenic RAS pathway activation promotes resistance to anti-VEGF therapy through G-CSF-induced neutrophil recruitment. Proc. Natl. Acad. Sci. USA *110*, 6079–6084.

Rakoff-Nahoum, S., and Medzhitov, R. (2009). Toll-like receptors and cancer. Nat. Rev. Cancer *9*, 57–63.

Ricciotti, E., and FitzGerald, G.A. (2011). Prostaglandins and inflammation. Arterioscler. Thromb. Vasc. Biol. *31*, 986–1000.

Rooney, M.S., Shukla, S.A., Wu, C.J., Getz, G., and Hacohen, N. (2015). Molecular and genetic properties of tumors associated with local immune cytolytic activity. Cell *160*, 48–61.

Rothwell, P.M., Wilson, M., Elwin, C.-E., Norrving, B., Algra, A., Warlow, C.P., and Meade, T.W. (2010). Long-term effect of aspirin on colorectal cancer incidence and mortality: 20-year follow-up of five randomised trials. Lancet *376*, 1741–1750.

Ruffell, B., Chang-Strachan, D., Chan, V., Rosenbusch, A., Ho, C.M.T., Pryer, N., Daniel, D., Hwang, E.S., Rugo, H.S., and Coussens, L.M. (2014). Macrophage IL-10 blocks CD8+ T cell-dependent responses to chemotherapy by suppressing IL-12 expression in intratumoral dendritic cells. Cancer Cell *26*, 623–637.

Schreiber, R.D., Old, L.J., and Smyth, M.J. (2011). Cancer immunoediting: integrating immunity's roles in cancer suppression and promotion. Science *331*, 1565–1570.

Sharma, P., and Allison, J.P. (2015). The future of immune checkpoint therapy. Science *348*, 56–61.

Talantov, D., Mazumder, A., Yu, J.X., Briggs, T., Jiang, Y., Backus, J., Atkins, D., and Wang, Y. (2005). Novel genes associated with malignant melanoma but not benign melanocytic lesions. Clin. Cancer Res. *11*, 7234–7242.

Tian, W., Zhao, Y., Liu, S., and Li, X. (2010). Meta-analysis on the relationship between nonsteroidal anti-inflammatory drug use and gastric cancer. Eur. J. Cancer Prev. *19*, 288–298.

Vesely, M.D., Kershaw, M.H., Schreiber, R.D., and Smyth, M.J. (2011). Natural innate and adaptive immunity to cancer. Annu. Rev. Immunol. *29*, 235–271.

Wang, D., and Dubois, R.N. (2010). Eicosanoids and cancer. Nat. Rev. Cancer *10*, 181–193.

Wenzel, J., Bekisch, B., Uerlich, M., Haller, O., Bieber, T., and Tüting, T. (2005). Type I interferon-associated recruitment of cytotoxic lymphocytes: a common mechanism in regressive melanocytic lesions. Am. J. Clin. Pathol. *124*, 37–48.

Willimsky, G., and Blankenstein, T. (2005). Sporadic immunogenic tumours avoid destruction by inducing T-cell tolerance. Nature *437*, 141–146.

Xu, L., Stevens, J., Hilton, M.B., Seaman, S., Conrads, T.P., Veenstra, T.D., Logsdon, D., Morris, H., Swing, D.A., Patel, N.L., et al. (2014). COX-2 inhibition potentiates antiangiogenic cancer therapy and prevents metastasis in preclinical models. Sci. Transl. Med. *6*, 242ra84.

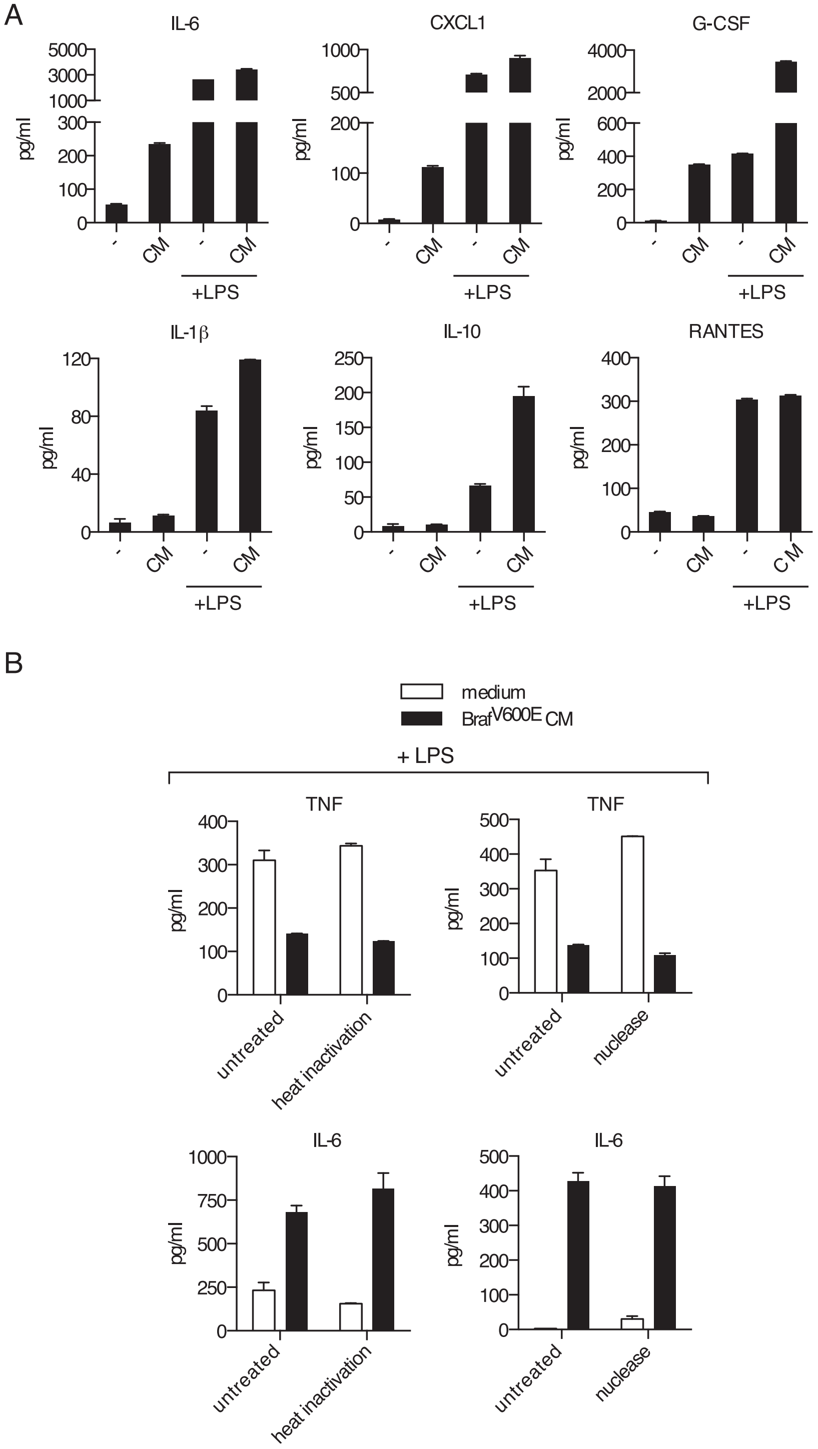

Figure S1. The Immunomodulatory Factor Secreted by Braf^{V600E} Cells Is Neither a Protein Nor a Nucleic Acid, Related to Figure 1

(A) BMMCs were cultured in presence or absence of 100 μl of CM from Braf^{V600E} cells plus or minus LPS (100 ng/ml). The concentration of IL-6, CXCL1, G-CSF, IL-1β, IL-10 or RANTES in the supernatant was determined after overnight culture.

(B) BMMCs were cultured in absence (medium) or presence (Braf^{V600E} CM) of 100 μl of untreated, heat-inactivated (20 min at 95°C) or nuclease-treated CM from Braf^{V600E} cells plus or minus LPS (100 ng/ml). The concentration of TNF (+LPS) and IL-6 (no LPS) in the supernatant was determined after overnight culture.

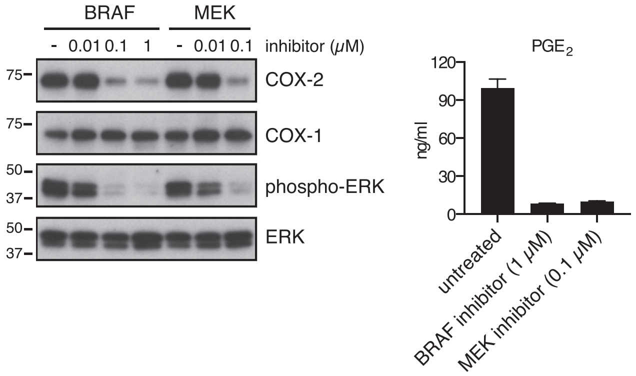

Figure S2. Braf or MEK Inhibition Reduces COX-2 Expression and PGE_2 Production in $Braf^{V600E}$ Cells, Related to Figure 1

$Braf^{V600E}$ melanoma cells were cultured for 48 hr in presence of a Braf inhibitor (PLX4720) or a MEK inhibitor (PD184352) at the indicated concentrations. The expression of COX-2, COX-1, phospho-ERK and ERK was determined by immunoblotting, left panels. For determination of PGE_2 in the supernatant (right panel), the medium was replaced at 24 hr.

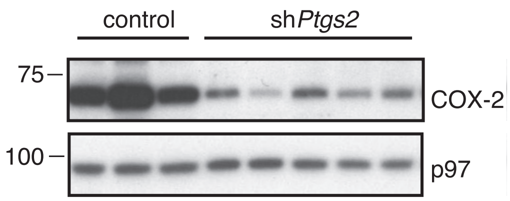

Figure S3. COX-2 Expression following *Ptgs2*-Specific Targeting Using shRNA, Related to Figure 1

Immunoblot of COX-2 in $Braf^{V600E}$ melanoma cells stably expressing various control or COX-2 specific shRNA constructs. p97 served as a loading control.

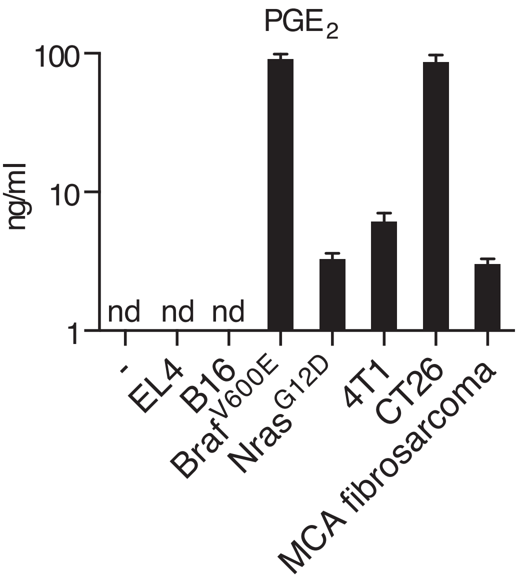

Figure S4. PGE$_2$ Production by Cancer Cell Lines, Related to Figure 1

EL4 thymoma, B16 melanoma, Braf^{V600E} melanoma, Nras^{G12D} melanoma, methylcholanthrene (MCA)-induced fibrosarcoma, 4T1 breast and CT26 colorectal cancer cell lines were cultured to confluency and the concentration of PGE$_2$ in the supernatant was determined by ELISA. nd, not detected.

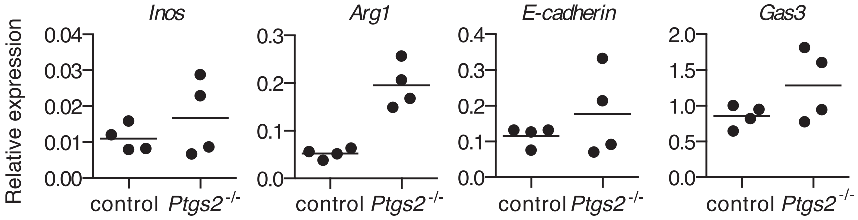

Figure S5. Expression of Markers Associated with M2 Polarization in Tumors Formed by *Ptgs2*$^{-/-}$ Cells, Related to Figure 2

WT mice were inoculated with control or *Ptgs2*$^{-/-}$ Braf^{V600E} cells and four days later the expression of *Inos, Arg1, E-cadherin* and *Gas3* mRNA was determined by qPCR in whole tumor homogenates. Relative expression of each gene was normalized to *hprt*. Each dot represents one independent tumor.

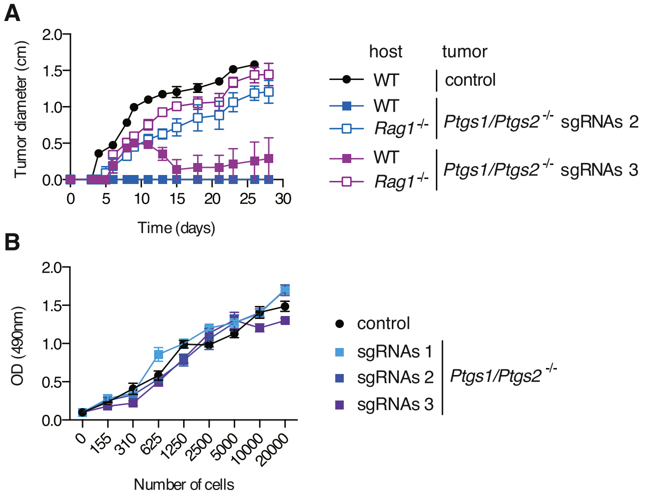

Figure S6. Immune-Dependent Control of Growth of Independent *Ptgs1/Ptgs2*$^{-/-}$ Clones, Related to Figure 4

(A) WT or *Rag1*$^{-/-}$ mice were inoculated with 10^5 control or two independent *Ptgs1/Ptgs2*$^{-/-}$ Braf^{V600E} melanoma cell lines (sgRNAs2 and sgRNAs3) generated using two combinations of sgRNAs targeting *Ptgs1* and *Ptgs2* that are different from the combination used to generate the *Ptgs1/Ptgs2*$^{-/-}$ Braf^{V600E} cells used in Figures 2, 3, 4, and 5 (sgRNAs 1). Tumor growth is presented as average tumor diameters ± SEM of three to five mice per group.

(B) The proliferative capacity of the three *Ptgs1/Ptgs2*$^{-/-}$ Braf^{V600E} melanoma cell lines used in this study (sgRNAs 1, sgRNAs 2 and sgRNAs 3) was compared to that of control COX-expressing Braf^{V600E} cells. The metabolic activity of the cells 72 hr after culture of the indicated number of cells was determined using a non-radioactive cell proliferation assay.

Cell

Supplemental Information

Cyclooxygenase-Dependent Tumor Growth through Evasion of Immunity

Santiago Zelenay, Annemarthe G. van der Veen, Jan P. Böttcher, Kathryn J. Snelgrove, Neil Rogers, Sophie E. Acton, Probir Chakravarty, Maria Romina Girotti, Richard Marais, Sergio A. Quezada, Erik Sahai, and Caetano Reis e Sousa

Supplemental Experimental Procedures

Mice. C57BL/6, *Rag1*$^{-/-}$, *Tap1*$^{-/-}$, *H2-Aa*$^{-/-}$ (*I-A*$\alpha^{-/-}$), *Batf3*$^{-/-}$ (kind gift from Kenneth M. Murphy, Washington University St. Louis), *Ifnar1*$^{-/-}$, Balb/c and nude mice were bred at Cancer Research UK in specific pathogen-free conditions.

Cancer cell lines. The MCA fibrosarcoma cell line was a kind gift from Robert D. Schreiber (Washington University St. Louis). COX-2 knockdown was performed using five non-overlapping shRNA hairpins targeting the coding sequence of murine *Ptgs2* (Ptgs2, NM_011198.2) selected via the RNAi Consortium of the Broad Institute (http://www.broadinstitute.org/rnai/public/). Three hairpins targeting eGFP were chosen as controls. Appropriate DNA oligonucleotides encoding these hairpins were cloned into the AgeI/EcoRI site of the lentiviral plasmid pLKO.1neo (obtained from Dr. S. Stewart via Addgene, plasmid 13425). Lentivirus was produced according to instructions provided by the Broad Institute (http://www.broadinstitute.org/rnai/public/resources/protocols). For generation of *Ptgs2*$^{-/-}$ Braf^{V600E} cells the *Ptgs2* sequence AGATGACTGCCCAACTCCCA**TGG** was targeted using the oligos Forward (Fw) CACCGGATGACTGCCCAACTCCCA and Reverse (Rv) AAACTGGGAGTTGGGCAGTCATCC. For sgRNAs 1 *Ptgs1/Ptgs2*$^{-/-}$ Braf^{V600E} cells the *Ptgs1* sequence TTACTATCCGTGCCAGAACC**AGG** was targeted using the oligos Fw CACCGTACTATCCGTGCCAGAACC and Rv AAACGGTTCTGGCACGGATAGTAC and the *Ptgs2* sequence TCCAATCCATGTCAAAACCG**TGG** using oligos Fw CACCGCCAATCCATGTCAAAACCG and Rv AAACCGGTTTTGACATGGATTGGC. For sgRNAs 2 *Ptgs1/Ptgs2*$^{-/-}$ cells the *Ptgs1* sequence CCGAGAAGTACTCATGCGCC**TGG** was

targeted using the oligos Fw CACCGCGAGAAGTACTCATGCGCC and Rv AAACGGCGCATGAGTACTTCTCGC and the *Ptgs2* sequence TCCAATCCATGTCAAAACCG**TGG** using oligos Fw CACCGCCAATCCATGTCAAAACCG and Rv AAACCGGTTTTGACATGGATTGGC. For sgRNAs 3 *Ptgs1/Ptgs2*$^{-/-}$ cells the *Ptgs1* sequence TTACTATCCGTGCCAGAACC**AGG** was targeted using the oligos Fw CACCGTACTATCCGTGCCAGAACC and Rv AAACGGTTCTGGCACGGATAGTAC and the *Ptgs2* sequence AGATGACTGCCCAACTCCCA**TGG** using oligos Fw CACCGGATGACTGCCCAACTCCCA and Rv AAACTGGGAGTTGGGCAGTCATCC. For *Ptgs2*$^{-/-}$ Nras^{G12D} melanoma cells and *Ptgs2*$^{-/-}$ CT26 cells the *Ptgs2* sequence TCCAATCCATGTCAAAACCG**TGG** was targeted using the oligos Fw CACCTCCAATCCATGTCAAAACCG and Rv AAACCGGTTTTGACATGGATTGGA. For *Ptgs1/Ptgs2*$^{-/-}$ 4T1 cells the *Ptgs1* sequence TTACTATCCGTGCCAGAACC**AGG** was targeted using the oligos Fw CACCGTACTATCCGTGCCAGAACC and Rv TCCAATCCATGTCAAAACCG**TGG** and the *Ptgs2* sequence using the oligos Fw CACCTCCAATCCATGTCAAAACCG and Rv AAACCGGTTTTGACATGGATTGGA.

For *Pges*$^{-/-}$ (*mPGES-1/mPGES-2*$^{-/-}$) Braf^{V600E} cells the *mPGES-1* sequence GGTCATCAAGATGTACGCGG**TGG** was targeted using the oligos Fw CACCGGTCATCAAGATGTACGCGG and Rv AAACCCGCGTACATCTTGATGACC and the *mPGES-2* sequence CCAGGCGGCGCGCCTTTCGT**GGG** using the oligos Fw CACCGCAGGCGGCGCGCCTTTCGT and Rv AAACCTTGCGGACCGCGGCGCCCC. Oligonucleotides corresponding to these guide sequences (PAM motif in bold) were cloned into the BbsI site of pX330, a bicistronic expression vector encoding both Cas9 and the sgRNA.

Plasmid pX330 was obtained from the Zhang lab via Addgene (plasmid 42230). $Braf^{V600E}$ cells were co-transfected in a 6-well plate using 1 μg pX330 and 1 μg pEGFP-C1 (Clontech) per well using Lipofectamine 2000 (Life technologies) according to the manufacturer's instructions. Twenty-four to 48 h post-transfection, GFP-positive cells were FACS sorted and replated at limiting dilution in order to pick individual colonies. Knockdown of COX-2 and ablation of COX-1 and/or COX-2 were also verified by immunoblotting using COX-1 and COX-2 specific antibodies (Cell Signaling). For determination of PGE_2 levels in vivo a fixed number of total tumor cell suspension was freeze-thawed and re-suspended in 100 μl. The concentration of PGE_2 in CM or in vivo was determined by ELISA (Cayman chemical). The proliferative capacity of cells was assessed using a non-radioactive cell proliferation assay (Promega) following manufacturer's instructions. The Braf inhibitor, PLX4720, was from Stratech and the MEK inhibitor, PD184352 from LC Labs. Anti-ERK and anti-phospho-ERK antibodies were from Cell Signaling. Anti-p97 antibody was from Fitzgerald Industries.

FACS analysis. For analysis of tumor infiltrating DCs, tumors were collected, cut into small pieces and digested with Collagenase IV (200 U/ml) and DNase I (0.2 mg/ml) for one hour at 37°C, washed with FACS buffer (PBS containing 2% FCS, 2 mM EDTA and 0.02% sodium azide) and pelleted. The following antibodies were purchased from BD Biosciences: anti-CD86-Fluorescein isothiocyanate (FITC) (clone GL1), anti-CD40-Phycoerythrin (PE) (clone 3/23), anti-CD16/32 (clone 2.4G2; Fc block) and anti-CD19-V450 (clone 1D3). The following antibodies were purchased from eBioscience: anti-MHCII I-A/I-E-APC-eFluor780 (clone M5/114.15.2), anti-CD11b-Alexa Fluor 700 or eFluor 605Nanocrystal (clone M1/70), anti-Ly6G-Alexa Fluor 700 (clone RB6-8C5) and anti-CD103 APC (clone 2E7). Anti-CD45.2-

PECy7 (clone 104) and anti-CD11c-PerCP/Cy5.5 (clone N418),were from Biolegend. Propidium iodide or Dapi were added to the final suspension to exclude dead cells before acquisition on a FACS-Calibur or LSR Fortessa (BD Biosciences) and analysis performed with Flowjo (Tree Star, Ashland, OR) software. Live cell counts were calculated from the acquisition of a fixed number of 10 μm latex beads (Coulter) mixed with a known volume of unstained cell suspension. Ex vivo intracellular IL-12 p40 staining was performed on isolated cells 6 hours following an intravenous injection of Brefeldin A (10 mg/g of body weight, Sigma-Aldrich). Tumor digestion was performed in presence of Brefeldin A (5 μg/ml). Cells were then stained for surface markers, fixed, permeabilized and then stained with anti-mouse PE-coupled anti-IL-12/23 p40 Ab (ebioscience, clone C17.8).

In vitro culture. Heat inactivation was performed by incubating CM before addition to DC cultures at 95°C for 20'. Nuclease treatment consisted in incubation of CM in presence of 2.5 Units/µl benzonase (Sigma) at 37°C for 30'. Synthetic PGE_2 was from Sigma. Capture anti-IL-12/23 p40 (BD Biosciences) and detection biotinylated anti-IL-12/23 p40 (BD Biosciences) antibodies were used for ELISA. Mouse TNF, MIP1α, IL-6, CXCL1, G-CSF, IL-1β, IL-10 and RANTES cytometric bead array flex sets were from BD Biosciences.

Quantitative PCR. Expression of *Ptgs2, Cd86, Tbx21, Ifng, Cxcl10, Cxcl11, Inos, Arg1, E-cadherin* and *Gas3* was determined by quantitative PCR using Invitrogen SYBR Green Fast master mix according to the manufacturer's instructions. Primers used: *Ptgs2*: Fw 5'-GAAGTGCCAATCCCCTAGCAA and Rv 5'- ATACCTGGAAGAACTTGATGCCC; *Cd86*: Fw 5'-ACAAAAAAAGCCACCCACAG and Rv 5'-ACGTGCAGGTCAAATTTATGC;

Tbx21: 5’-CAACAACCCCTTTGCCAAAG and Rv 5’- TCCCCCAAGCAGTTGACAGT; *Ifng*: Fw 5’-GGATGCATTCATGAGTATTGC and Rv 5’-CCTTTTCCGCTTCCTGAGG; *Cxcl10*: Fw 5’- TCAAGCCATGGTCCTGAGACAA and Rv 5’-CGCACCTCCACATAGCTTACAG; *Cxcl11*: Fw 5’-CCTGGGAACGTCTGACTGTG and Rv 5’- TCTGCAGCCTGGTAATACGTG; *Inos*: Fw 5’-CAGCTGGGCTGTACAAACCTT and Rv 5’-CATTGGAAGTGAAGCGTTTCG; Arg1: Fw 5’-GAACACGGCAGTGGCTTTAAC and Rv 5’-TGCTTAGCTCTGTCTGCTTTGC; E-cadherin: Fw 5’-ACTTGGGGACAGCAACATCA and Rv 5'-GGGTTTAAATCGGCCAGCAT; Gas3: Fw 5’-GTAATGGACACACGACTGATC and Rv 5’-GGAGTAGTCAGTGTTGACATG. HPRT: Fw 5’-TCAGTCAACGGGGGACATAAA and Rv 5’-GGGGCTGTACTGCTTAACCAG as normalization control was used to assess relative gene expression. Expression of *Il6* (Mm00446190_m1), *Cxcl1* (Mm04207460_m1), *Il12b* (Mm99999067_m1), *Tnf* (Mm00443258_m1), *Ifit1* (Mm00515153_m1) and *Ifit2* (Mm00492606_m1) was determined by relative standard curve method using Taqman reagents according to manufacturer’s instructions and normalized to GapDH (Mm99999915_g1) expression (Applied Biosystems).

Human microarray dataset analysis. Data were analyzed using Bioconductor 2.13 running on R 3.0.2 (available from www.R-project.org). Probeset expression measures were calculated using the Affymetrix package’s Robust Multichip Average (RMA) default method. The GSE3189 dataset comprised of 45 melanoma samples and were ranked from lowest to highest using COX-2 (PTGS2) expression. The top and bottom 10 samples (~20%) were extracted from the dataset and used to look for enrichment of gene sets using Gene set enrichment analysis (GSEA)(Subramanian et al., 2005).
